# Discovery and biochemical characterization of enzymes completing the 4-hydroxyphenylacetate pathway in *Acinetobacter* baumannii TH

**DOI:** 10.1016/j.jbc.2025.110917

**Published:** 2025-11-05

**Authors:** Wachirawit Chinantuya, Kittipop Kungchuai, Pimchai Chaiyen, Somchart Maenpuen, Ruchanok Tinikul

**Affiliations:** 1 Department of Biochemistry and Center for Excellence in Protein and Enzyme Technology, Faculty of Science, Mahidol University, Bangkok, Thailand; 2 School of Biomolecular Science and Engineering, Vidyasirimedhi Institute of Science and Technology (VISTEC), Rayong, Thailand; 3 Department of Biochemistry, Faculty of Science, Burapha University, Chonburi, Thailand

**Keywords:** *p*-hydroxyphenylacetate, aromatic degradation, isomerase, decarboxylase, hydratase, protein interaction, *Acinetobacter baumannii* TH

## Abstract

A metabolic pathway for degrading 4-hydroxyphenylacetate (4-HPA) is crucial for environmental and pathogenic microbes to assimilate aromatic compounds. The 4-HPA degradation pathway in *Acinetobacter baumannii* TH comprises multiple reactions that are not fully understood. Enzymes involved in the first two steps (a two-component 4-HPA-3-hydroxylase, and 3,4-dihydroxyphenylacetate 2,3-dioxygenase) and the last two steps (4-hydroxy-2-keto-heptane-1,7-dioate aldolase and succinic semialdehyde (SSA) dehydrogenase (SSADH)) have been identified and studied, while the enzymes functioning in the middle of the pathway remain uncharacterized. Here, we identified products associated with individual enzymes including 5-carboxymethyl-2-hydroxymuconate-semialdehyde (CHMS) dehydrogenase (CHMSD), 5-carboxymethyl-2-hydroxymuconate (CHM) isomerase (CHMI), five-oxo-pent-3-ene-1,2,5-tricarboxylate (OPET) decarboxylase (OPETD), 2-hydroxy-hept-2,4-diene-1,7-dioate (HHDD) isomerase (HHDDI) and two-oxo-hept-3-ene-1,7-dioate (OHED) hydratase (OHEDH). We used enzymatically synthesized OPET (a tri-acid) to probe the decarboxylation step and found that the highest decarboxylation efficiency was achieved when OPETD, CHMI, and HHDDI were all present in the reaction. We demonstrated that CHMI was responsible for tri-acid tautomerization, while the protein-protein interactions between OPETD and HHDDI enhanced the decarboxylation by OPETD to generate OHED (a di-acid). OHEDH is distinct from other hydratases in that it requires Mn^2+^ as a cofactor. Notably, besides CHMS, CHMSD can use SSA, a substrate of SSADH, suggesting that CHMSD can substitute for SSADH to generate succinate for cellular utilization. Our studies herein completely assigned the catalytic functions of all enzymes in the 4-HPA degradation pathway. The knowledge gained will be valuable for developing inhibitors targeting enzymes unique to pathogenic microbes or for constructing cascade reactions to convert lignin-derived compounds into valuable biochemicals.

Aromatic degradation pathways are prevalent in numerous aerobic bacteria, enabling these organisms to utilize aromatic compounds as sources of carbon and energy (1). Notably, enzymes that degrade aromatic compounds, particularly phenolic compounds, are not found in animal or human metabolic pathways, rendering them attractive targets for drug development (2, 3). In the natural habitat, degradation of aromatic compounds is crucial for the global carbon cycle (4) and serves as a vital mechanism for removing organic contaminants from polluted areas (5). As innovative research biorefineries and synthetic biology focuses on the use of biomass as renewable carbon feedstocks, the enzymatic reactions in aromatic degradation pathways offer promising strategies for the valorization of lignin- and waste-derived aromatic compounds into value-added biochemicals, fuels, and energy (6, 7).

Bacterial degradation of 4-hydroxyphenylacetate (4-HPA) enables aerobic bacteria to convert this compound into central metabolic intermediates such as succinic acid (SA) and pyruvate (PYR) *via* oxygen insertion, aromatic ring cleavage, isomerization, oxidation, and carbon-carbon bond cleavage to meet the energy and metabolic needs for cell growth (8, 9). The gene cluster involved in 4-HPA degradation has been investigated in several aerobic bacteria such as *Acinetobacter* sp. (8, 10), *Klebsiella pneumoniae* (11), *Burkholderia xenovorans* LB400 (12), *Pseudomonas putida* F6 (13), *Pseudomonas aeruginosa* PAO1 (14), *Salmonella dublin* (10) and *Escherichia coli* W and C (15, 16). Although these operons differ in gene number and arrangement, they are typically organized into two adjacent operons: (i) the upper *hpa* cluster or HPA hydroxylase operon, encoding a two-component flavin-dependent hydroxylase that hydroxylates 4-HPA to produce 3,4-dihydroxyphenylacetate (3,4-DHPA), and (ii) the *meta*-cleavage operon, which encodes enzymes that convert 3,4-DHPA into SA and PYR, as well as regulatory proteins that control expression of genes in the operons (15).

In *Acinetobacter baumannii,* the nine sequential enzymatic steps proposed for 4-HPA degradation (Fig. 1) include a two-protein (reductase and oxygenase) component 4-HPA-3-hydroxylase (HPAH), 3,4-dihydroxyphenylacetate 2,3-dioxygenase (3,4-DHPAO), 5-carboxymethyl-2-hydroxymuconate-semialdehyde dehydrogenase (CHMSD), 5-carboxymethyl-2-hydroxymuconate isomerase (CHMI), 5-hydroxypenta-2,4-diene-1,2,5-tricarboxylate decarboxylase (OPETD), 2-hydroxy-hept-2,4-diene-1,7-dioate isomerase (HHDDI), two-oxo-hept-3-ene-1,7-dioate hydratase (OHEDH), 4-hydroxy-2-keto-heptane-1,7-dioate aldolase (HKHDA), and succinic semialdehyde dehydrogenase (SSADH), which together produce PYR and SA intermediates (10). Some enzymes and their homologues, such as HPAH (an oxygenase component) (17, 18, 19) and 3,4-DHPAOs (20, 21), SSADH (22) and HKHDAs (23, 24), have been extensively studied in *A. baumannii* and other bacteria, such as *P. aeruginosa* and *E. coli*, for their structures, functions, and reaction mechanisms, as well as their potential applications as biocatalysts to synthesize valuable compounds. Notably, while upstream enzymes (HPAH (25) and 3,4-DHPAO (20)) and downstream enzymes (HKHDA (23) and SSADH (22, 26)) of the pathway have been well-studied (Fig. 1, *gray steps*), there is a notable lack of information regarding enzymes in the middle of the pathway, including CHMSD, CHMI, OPETD, HHDDI, and OHEDH (Fig. 1, *blue steps*). This knowledge gap motivated our investigation into the biochemical, biophysical, and catalytic properties of these five enzymes.

**Figure 1:**
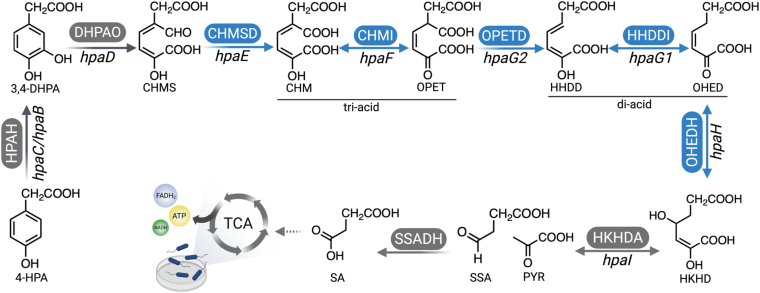
**The proposed enzymatic reactions in the 4-HPA degradation pathway.** The enzymes in the first-two step (two-component HPAH and 3,4-DHPAO) and last-two step (HKHDA and SSADH) were extensively studied in structure and function (*gray arrow*), while the enzymes in the middle of this pathway are characterized in this study (*blue arrow*).

In the middle pathway, CHMSD from *E. coli* C has been proposed to catalyze the NAD ^+^ -dependent conversion of 5-carboxymethyl-2-hydroxymuconate-semialdehyde (CHMS) to 5-carboxymethyl-2-hydroxymuconate (CHM) (27). This enzyme contains conserved residues typical of aldehyde dehydrogenases (28). However, CHMSD information remains limited, with only the CHMSD in *E. coli* C cell extract being documented (27). CHMI from *E. coli* B is proposed to catalyze the isomerization of CHM to form a tricarboxylic acid (tri-acid) or OPET. The initial study from native *E. coli* cell extract enzyme demonstrated that CHM can undergo spontaneous isomerization; however, this conversion occurs more rapidly in the presence of CHMI (29). Nevertheless, the catalytic and biochemical properties of CHMI have not been reported. The decarboxylase enzyme (OPETD) in the *E. coli* W *hpa* operon is encoded by *hpaG* (30, 31) and identified as a bifunctional enzyme catalyzing decarboxylation and isomerization reactions (31). Analyses of the HpcE from *E coli* C sequence and structure have identified two structural domains, the N- (isomerase) and C-terminal (decarboxylase) domains, exhibiting similar folding patterns (32, 33). However, this domain-specific functional assignment was solely based on crystal structure data without experimental validation. Notably, among the identified *hpa* operons, the bilobed architecture was only observed in HpaG from *E. coli* W (also known as HpcE in *E. coli* C) (32) and HpaG from *S. dublin* (10)). Analyses of *hpa* operons from other bacteria (11, 34, 35) revealed a tandem arrangement of two genes, *hpaG1* and *hpaG2*, encoding two separate proteins (35). The biological significance of this major difference remains to be explored. For the subsequent hydration step catalyzed by OHEDH, it has been reported that the hydratase activity in other aromatic degradation pathways depends on divalent metal ions to achieve a fully active enzyme for substrate conversion (36, 37, 38). However, the actual functional role of metal ions through the reaction mechanisms has not been elucidated.

In this study, we fully elucidated the cascade reactions of the 4-HPA degradation pathway in *A. baumannii* TH and characterized the catalytic and biochemical properties of five putative enzymes (CHMSD, CHMI, OPETD, HHDDI, and OHEDH) involved in isomerization, decarboxylation, and hydration steps (Fig. 2). Their functional roles were assigned based on the identifications of products from individual reactions. Our findings demonstrate that the presence of the isomerases CHMI and HHDDI enhances the enzymatic efficiency of OPETD in decarboxylating a tri-acid intermediate through protein-protein interactions; a stable complex between OPETD and HHDDI was detected using the size-exclusion chromatographic (SEC). The resulting di-acid product can be further hydrated by Mn^2+^-dependent OHEDH. Furthermore, we discovered that CHMSD also function as a succinic semialdehyde (SSA) dehydrogenase (SSADH) by dehydrogenating SSA—a non-native substrate for CHMSD. The ability of CHMSD to compensate for SSADH function explains a long-standing puzzle regarding the absence of the *ssadh* gene in the 4-HPA degradation operon of many bacteria. The findings from this study provide mechanistic insights into how various enzymes in the HPA degradation pathway function and coordinate to metabolize aromatic substrates.

**Figure 2:**
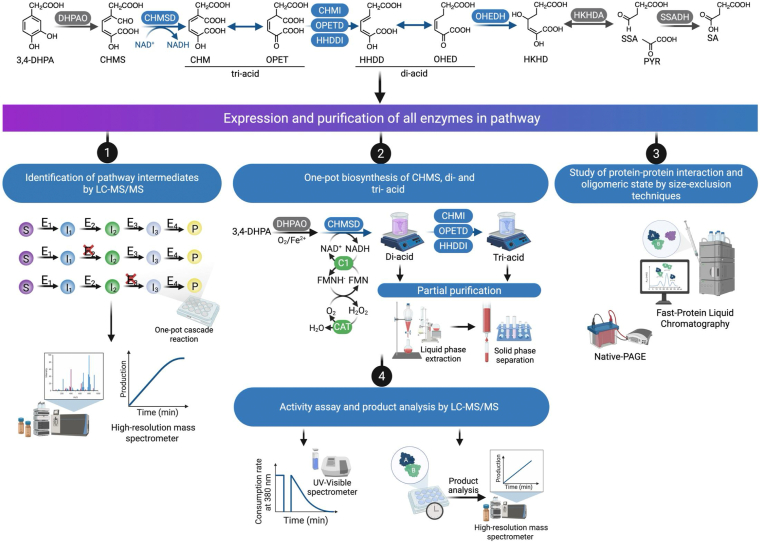
**The overall workflow of the experiments to study the complete degradation pathway of 4-HPA from *A. baumannii* TH.** The experimental setup consists of the expression and purification of all enzymes involved in the pathway, identification of pathway intermediates using LC-MS/MS, establishment of one-pot biosynthesis of CHMS, di-acid, and tri-acid, as well as the study of protein-protein interactions and oligomeric states through size exclusion techniques. Additionally, activity assays and product analyses are conducted using LC-MS/MS.

## Results

### Enzyme expression and purification

The genes encoding individual enzymes including CHMSD, CHMI, OPETD, HHDDI, and OHEDH, from *A. baumannii* TH were amplified and cloned into the pET-11a vector at the *Nde*I and *BamH*I restriction sites. Plasmids for overexpressing the C1 reductase of the two-component flavin-dependent hydroxylase, 3,4-DHPAO, SSADH, and HKHDA were obtained from previous studies (10, 21, 22, 25). The 4-HPA degrading enzymes from *A. baumannii* TH, including CHMSD, CHMI, OPETD, HHDDI, and OHEDH were overexpressed without any affinity tag under the control of an inducible T7 promoter using auto-induction media in *E. coli* BL21(DE3). Cells overexpressing these enzymes were cultured overnight at 25 °C (see Experimental procedures). The purification process involved polyethylenimine (PEI) and ammonium sulfate ((NH_4_)_2_SO) precipitations, followed by anion-exchange and hydrophobic interaction chromatography, yielding the purified enzymes in the range of 50 to 118 mg per liter of bacterial culture with >95% purity as judged by SDS-PAGE (Fig. S1 and Table S1).

### Identification of products from one-pot enzymatic reactions of 4-HPA-degrading enzymes

The 4-HPA degradation operon in *A. baumannii* TH differs from the *hpa* operon in *E. coli W*. In *E. coli* W, the *hpaG* gene encodes a bifunctional decarboxylase-isomerase enzyme, whereas in *A. baumannii TH*, similar functions are encoded by two distinct genes, *hpcG1* and *hpcG2*. In addition, the *hpa* operon of *A. baumannii* TH is the only reported version containing an additional *ssadh* gene, which encodes SSADH, catalyzing the conversion of succinic semialdehyde into SA in the final step of the pathway (10, 22). These differences raised questions about how this gene (enzyme) arrangement achieves the same pathway function, especially in operons lacking the *ssadh* gene and how SSADH activity is compensated.

**Figure 3:**
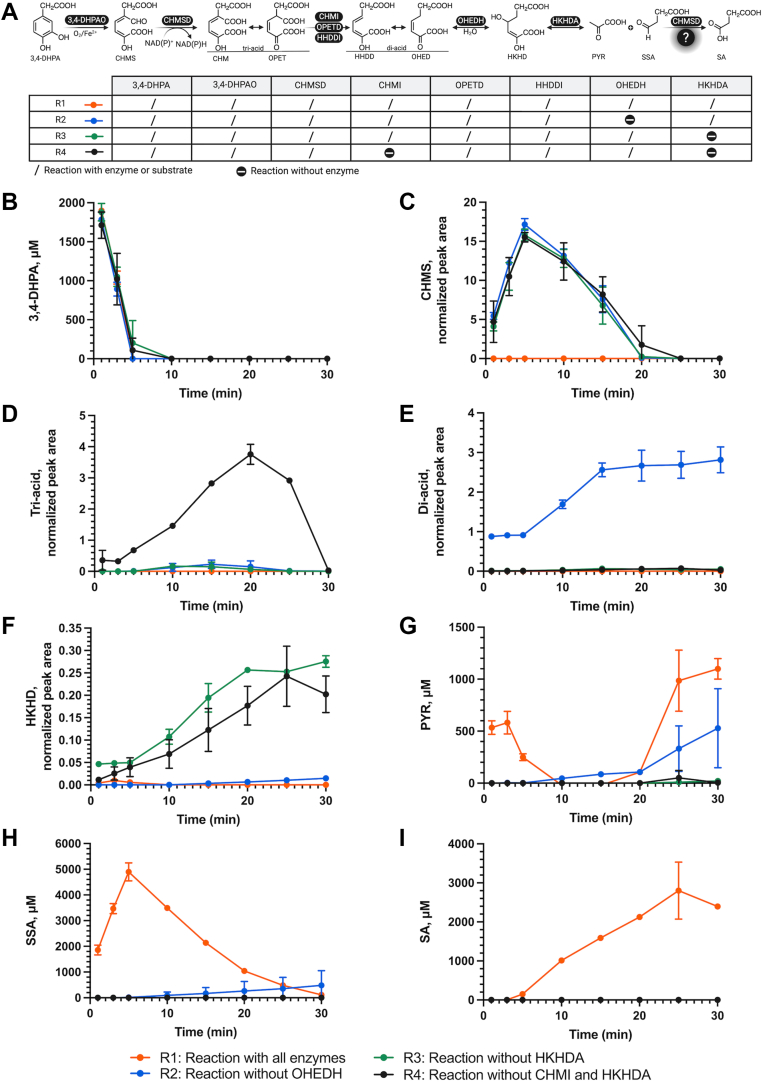
**The identification of intermediates in 4-HPA degradation pathway using one-pot enzymatic assays.***A*, a reaction scheme illustrating the enzymatic reaction order and different colors of solid lines representing different one-pot enzymatic reaction setups. R1 represents the enzymatic reaction containing 3,4-DHPAO, CHMSD, CHMI, OPETD, HHDDI, OHEDH, and HKHDA (*orange*). R2 denotes the cascade enzymatic reaction omitting OHEDH (*blue*). R3 refers to the cascade enzymatic reaction subtracting HKHDA (*green*), and R4 refers the cascade reaction lacking of HHDDI and HKHDA (*black*), The predicted m/z of each product intermediate, (*B*) 3,4-DHPA (m/z 167.0), (*C*) CHMS (m/z 199.0), (*D*) tri-acids (m/z 215.0), (*E*) di-acids (m/z 171.0), (*F*) HKHD (m/z 189.0), (*G*) pyruvate (m/z 87.0), (H) SSA (m/z 101.0), and (*I*) SA (m/z 117.0), was analyzed by a negative ion mode in LC-MS/MS and the corresponding peak area was normalized with pimelic acid internal standard.

To investigate this, we analyzed pathway intermediates formed during the *in vitro* enzymatic cascade conversion of 3,4-DHPA using one-pot reactions (Fig. 3*A*). The predicted m/z of all pathway intermediates, including CHMS, tri-acids (CHM/OPET), di-acids (HHDD/OHED), HKHD, PYR, SSA, and SA (Table S2), were monitored and analyzed by High Performance Liquid Chromatography system coupled with high-resolution mass spectrometry (HPLC-MS/MS).

In reaction R1 (Fig. 3, *orange line*), 3,4-DHPA was rapidly depleted within 5 min (Fig. 3*B*), while most intermediates—CHMS, tri-acids, di-acids, and HKHD—accumulated only at low levels (Fig. 3, *C*–*F*). Only the HKHDA reaction products, PYR (Fig. 3*G*) and SSA (Fig. 3*H*), were generated along with 3,4-DHPA decrease, confirming that the cascade conversion from 3,4-DHPA to PYR and SSA was complete. It should be noted that there was a fluctuation in PYR concentrations detected (Fig. 3*G*). This could be explained that a byproduct H_2_O_2_, generated from flavin reductase-catalyzed NAD^+^/NADH regenerating system used in a one-pot enzymatic assay condition (see Experimental procedures section), could react with PYR *via* nucleophilic addition to form an unstable hydroperoxide adduct (39, 40), which may cause the degradation and fluctuation of pyruvate. This was confirmed by our investigation which found that PYR concentration rapidly decreased within 2 min and gradually decayed afterward in the presence of H_2_O_2_ (Fig. S2). SSA levels gradually decreased after 5 min (Fig. 3*H*), whereas SA levels continued to rise, even in the absence of SSADH (Fig. 3*I*). This observation suggests that other enzymes, potentially CHMSD, can catalyze the oxidation of SSA to SA in the absence of SSADH. Furthermore, the low accumulation of intermediates and rapid substrate turnover across the pathway indicates that the conversion from 3,4-DHPA to PYR and SA is both efficient and thermodynamically favorable.

In reaction R2 which lacked OHEDH (Fig. 3, *blue line*), 3,4-DHPA was still consumed (Fig. 3*B*), and CHMS accumulated within 5 min (Fig. 3*C*). In contrast, other products remained at very low levels. From 5 to 15 min, CHMS levels slowly declined, accompanied by a modest increase in tri-acids (Fig. 3*D*) and a significant rise in di-acids (Fig. 3*E*). However, the remaining intermediates did not accumulate (Fig. 3, *F*–*I*). These data implied that tri-acids, generated from the dehydrogenation of CHMS, underwent decarboxylation to yield di-acids readily, thus resulting in low accumulation of tri-acids. The lack of OHEDH in this R2 reaction prevented further hydration steps from occurring, thus resulting in the accumulation of di-acids and no subsequent formation of HKHD, PYR, and SSA. These results confirm that OHEDH is crucial for the hydration of di-acids in the pathway.

In the reaction R3 which lacked HKHDA (Fig. 3, *green line*), it was observed that, similar to reactions R1 and R2, 3,4-DHPA was rapidly consumed within 5 min (Fig. 3*B*), which aligned with the increase in CHMS (Fig. 3*C*), while other intermediates appeared in low amounts (Fig. 3, *D*–*I*). After 5 min, only HKHD product intermediate formed and then accumulated until 20 min, while CHMS slowly declined. Unlike in reaction R2, no accumulation of di-acids was observed (Fig. 3*E*), suggesting that the di-acids were rapidly converted into HKHD. This observation supports the proposed reaction order in the 4-HPA degradation pathway. The lack of HKHDA blocked the production of PYR and SSA when compared to the reaction R1, confirming the crucial role of HKHDA in catalyzing the C-C bond cleavage of HKHD to generate PYR and SSA.

Similar to reactions R1, R2, and R3, reaction R4 (Fig. 3, *black line*), which lacked CHMI and HKHDA, showed a decrease in 3,4-DHPA (Fig. 3*B*) with an increase in CHMS (Fig. 3*C*) within 5 min. Only two pathway intermediates, tri-acids (Fig. 3*D*) and HKHD (Fig. 3*F*), were clearly detected after 5 min, while other pathway intermediates were not significantly observed. Owing to the absence of HKHDA, HKHD accumulated until 25 min and then plateaued, while tri-acids increased until 20 min and subsequently declined. Even though CHMI was absent, the presence of the downstream intermediate HKHD (Fig. 3*F*) might be attributed to the non-enzymatic spontaneous isomerization of CHM to OPET, which has been reported to occur at a very low rate (29). Thus, tri-acid decarboxylation could still proceed, although the decarboxylation rate in reaction R4 was slower than in R3. These results indicate that CHMI is important for accelerating the decarboxylation step and support the proposed reaction order in the 4-HPA degradation pathway.

Notably, except in reaction R1, the CHMS product/intermediate was accumulated maximally after 5 min, despite CHMSD being present in all reactions (Fig. 3*C*; *blue, green and black lines*). We therefore speculate that the CHMSD reaction may be slow and is the rate-limiting step for the overall 4-HPA degradation pathway.

### CHMSD can catalyze dehydrogenation of SSA and thus can compensate for SSADH function

As results from the previous section showed, SA could be produced in the cascade reaction R1 in the absence of SSADH (Fig. 3*I*, *orange line*). We hypothesized that CHMSD may be able to compensate for SSADH activity. We thus investigated and compared CHMSD activity using SSA and its putative substrate, CHMS. In addition, activity assays of SSADH with both substrates were also carried out for comparison.

**Figure 4:**
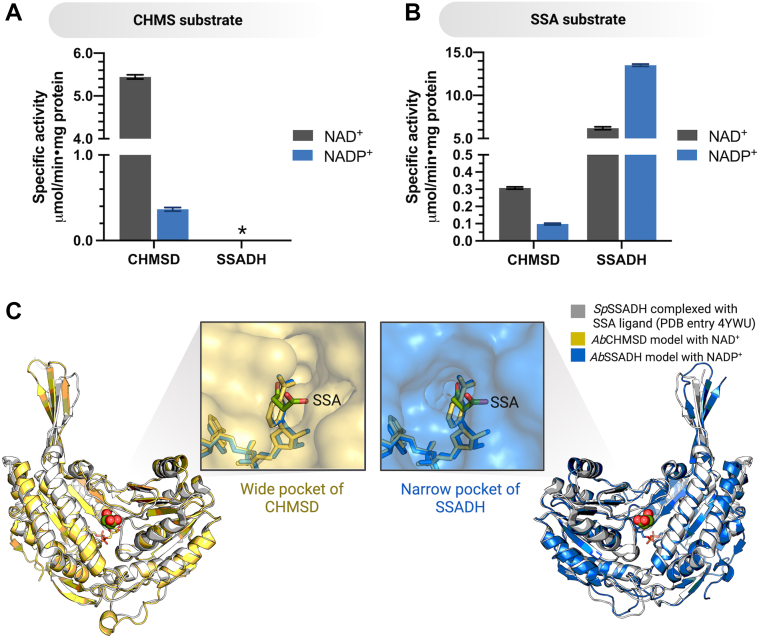
**The CHMSD and SSADH activities towards CHMS and SSA substrates.** The specific activity of CHMSD and SSADH using CHMS (*A*) and SSA (*B*) substrates in the presence of NAD^+^ (*gray*) and NADP^+^ (*blue*). *C*, the molecular surface models of CHMSD and SSADH with the incorporation of NAD(P)^+^, generated by Alphafold3, superimposed with homologous SSADH enzyme complexed with SSA ligand from *Streptococcus pyogenes* MGAS1882 (PDB entry 4YWU) and visualized using PyMOL showed the active site cavity of CHMSD being wider than that of SSADH. The *asterisk* indicates no CHMS oxidation activity.

The results in Figure 4 showed that CHMSD could catalyze the dehydrogenation of both SSA and CHMS when NAD^+^ and NADP^+^ were present. In contrast, SSADH could not use CHMS as a substrate (Fig. 4*A*) because it is specific only to its native substrate, SSA (Fig. 4*B*). These results support the previous findings that a full enzymatic cascade reaction lacking SSADH can produce SA because of CHMSD activity (Fig. 3*I*, *orange line*). To understand the structural basis of this observation, modeled three-dimensional structures of both CHMSD and SSADH (Fig. S3) were generated by AlphaFold3 (41) using various semialdehyde dehydrogenases homologs as templates (PDB entry 2D4E as a template for CHMSD and PDB entries 4YWU (42), 5VBF, 3JZ4 (43), and 4V6H as templates for SSADH) and superimposed with the structure of a binary complex SSADH:SSA (4YWU, from *Streptococcus pyogenes*) by PyMOL. The results revealed that both CHMSD and SSADH showed similar folding with 38.06% sequence identity. However, the active site cavity of CHMSD is wider than that of SSADH (Fig. 4*C*), explaining its ability to accommodate both CHMS and SSA. In contrast, the SSADH active site pocket is narrow, thus lacking the ability to bind CHMS. The catalytic residues important for aldehyde dehydrogenase catalysis, *i.e.,* cysteine and glutamate, are conserved in both CHMSD and SSADH (Fig. S4*A*) (22, 26). The analysis of active site residues relative to these catalytic residues also showed that SSA binds with the carbonyl aldehyde located near the catalytic cysteine (Fig. S4*B*). It should be emphasized that CHMSD prefers to use NAD^+^ over NADP^+^ in reactions utilizing both substrates, CHMS and SSA (Fig. 4, *A* and *B*), while SSADH prefers to use NADP^+^ in the SSA-utilizing reaction. These results suggest that the active site environment for accommodating both semialdehyde and electron-acceptor substrates differs between the two enzymes.

### Synergetic interactions between CHMI, OPETD, and HHDDI for efficient catalysis of tri-acid decarboxylation

**Figure 5:**
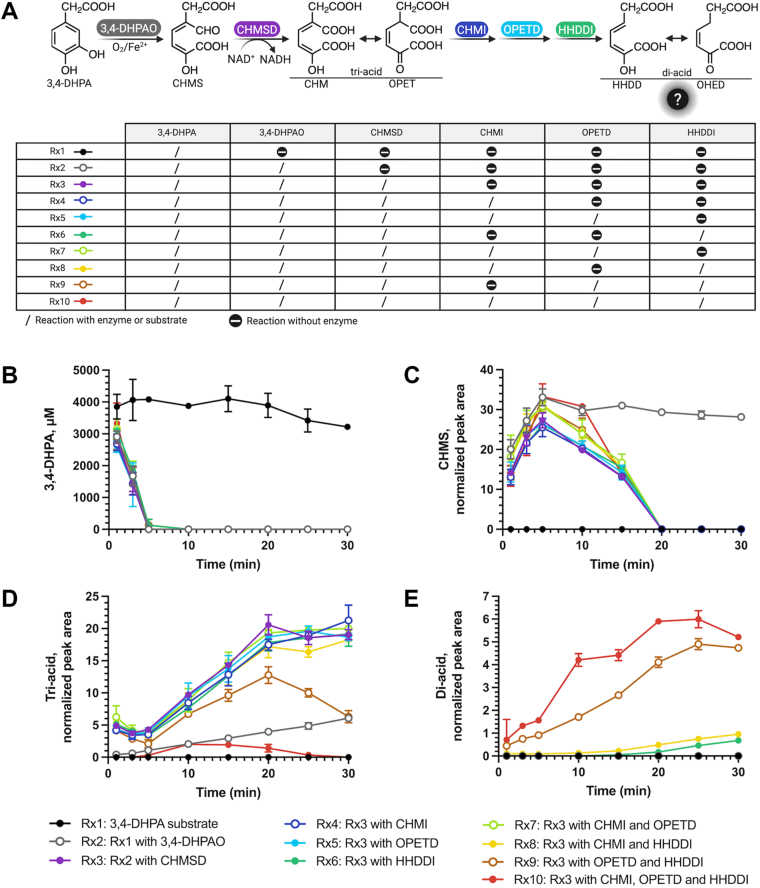
**Detection of tri-acids and di-acids formed during one-pot enzymatic cascade 3,4-DHPA ring-cleavage reactions.***A*, the schematic representation of ten different setups of one-pot enzymatic cascade 3,4-DHPA ring-cleavage reaction, which include the reactions R x 1 adding only a starting substance 3,4-DHPA (*black*), R x 2 containing 3,4-DHPAO (*gray*), R x 3 containing 3,4-DHPAO and CHMSD (*purple*), R x 4 containing 3,4-DHPAO, CHMSD, and CHMI (*blue*), R x 5 containing 3,4-DHPAO, CHMSD, and OPETD (*light blue*), R x 6 containing 3,4-DHPAO, CHMSD, and HHDDI (*green*), R x 7 containing 3,4-DHPAO, CHMSD, CHMI, and OPETD (*light green*), R x 8 containing 3,4-DHPAO, CHMSD, CHMI, and HHDDI (*yellow*), R x 9 containing 3,4-DHPAO, CHMSD, OPETD, and HHDDI (*brown*), and R x 10 including all enzymes (*red*). The product intermediates, such as (*B*) 3,4-DHPA, (*C*) CHMS, (*D*) tri-acids (CHM/OPET), and (*E*) di-acids (HHDD/OHED) were monitored for their predicted m/z values by a high-resolution LC-MS/MS. Their peak areas were normalized with the internal standard pimelic acid.

Results from the previous section demonstrated that, in the absence of OHEDH, the di-acid product intermediates accumulated during a one-pot enzymatic cascade reaction (Fig. 3, *A* and *E*, *blue lines*), indicating that the decarboxylation of tri-acid intermediates occurred efficiently. Therefore, we hypothesized that three upstream enzymes, including CHMI, OPETD, and HHDDI, may be involved in the tri-acid decarboxylation process. To dissect the catalytic function of these enzymes in tri-acid decarboxylation, 10 different one-pot enzymatic cascade setups starting from 3,4-DHPA substrate were performed (Fig. 5*A*). Five enzymes were included in these one-pot setups including 3,4-DHPAO, CHMSD, CHMI, OPETD, and HHDDI together with the auxiliary NAD^+^-regenerating C1-flavin reductase system.

**Figure 6:**
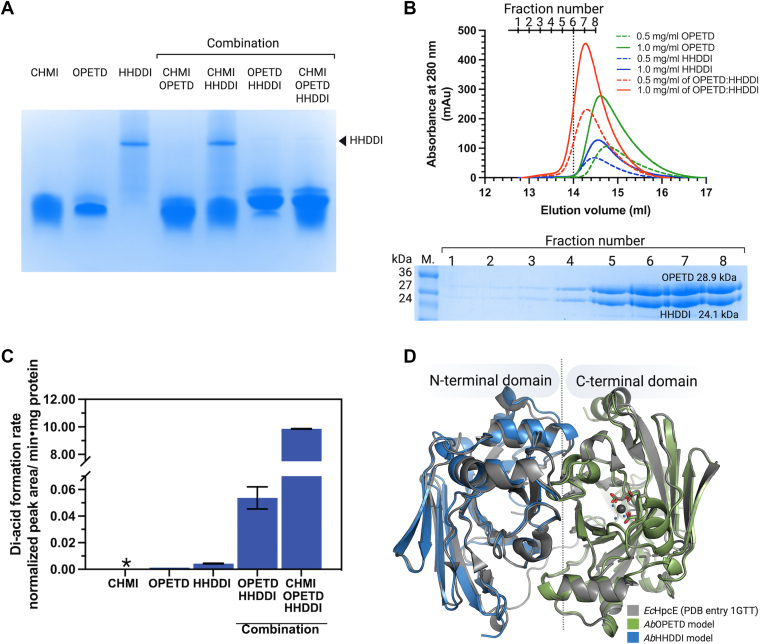
**The protein assembly of OPETD and HHDDI to form a binary complex.***A*, the Native-PAGE (10 μg protein for each lane) for analyzing the protein-protein interaction of enzymes involving in tri-acid decarboxylation, including CHMI, OPETD and HHDDI. *B*, The FPLC chromatograms of the eluted proteins representing OPETD, HHDDI, and OPETD- HHDDI complex. *C*, the specific tri-acid decarboxylation activities of OPETD, HHDDI, CHMI, OPETD- HHDDI complex, and OPETD- HHDDI together with CHMI. *D*, the modeled monomeric structures of OPETD (*green*) and HHDDI (*blue*) from *A. baumannii* TH generated by AlphaFold3 superimposed with an *Ec*HpcE bi-functional isomerase/decarboxylase homolog, which comprised of N-terminal (equivalent to *Ab*HHDDI) and C-terminal (equivalent to *Ab*OPETD) domains. The asterisk indicates no tri-acid decarboxylation activity.

The results revealed that without any enzyme (Rx1), 3,4-DHPA remained constant over time (Fig. 5*B*, *black line*), while it rapidly decreased in reactions Rx2–Rx10 within 5 min. Simultaneously, CHMS was produced and subsequently decayed in reactions Rx3–Rx10 (Fig. 5*C*) but remained unchanged in reaction Rx2 owing to lack of CHMSD (Fig. 5*C*, *gray line*). The CHMS decay (Fig. 5*C*) correlated with the substantial increase of tri-acids (CHM/OPETD) until 20 min in the reactions Rx3–Rx9 (Fig. 5*D*). However, only in Rx9 did tri-acids decrease after 20 min (Fig. 5*D*, *brown line*), whereas in Rx3–Rx8, tri-acids continued to accumulate. Notably, small amounts of tri-acids formed in Rx2 (Fig. 5*D*, *gray line*) may result from non-enzymatic oxidation. The data indicate that the presence of only CHMI, OPETD, or HHDDI (Rx4–Rx6), caused high accumulation of tri-acids, suggesting that decarboxylation could not occur with only one enzyme. Similarly, in Rx7 (CHMI+OPETD) and Rx8 (CHMI+HHDDI), tri-acids also accumulated (Fig. 5*D*), and di-acid production remained low (Fig. 5*E*). The co-addition of OPETD and HHDDI without adding CHMI in Rx9 (Fig. 5*D*, *brown line*) revealed that tri-acids accumulated for a while and then declined to yield the following di-acids. In Rx10, where all enzymes relevant to decarboxylation of tri-acids were included, tri-acids showed a slight increase at 10 min (Fig. 5*D*, *red line*), followed by a significant decrease, correlating with a considerable increase in di-acids (Fig. 5*E*). These results indicate that at least OPETD and HHDDI are needed for efficient decarboxylation of tri-acids (Fig. 5, *brown line*), and that optimal decarboxylation of tri-acids requires CHMI, OPETD, and HHDDI, working together. The data suggest that CHMI, annotated as a tri-acid isomerase, enhances the decarboxylation process more rapidly (see later in Fig. 6*C*) and that CHMI-catalyzed isomerization of tri-acids is an important step for achieving the highest decarboxylation efficiency.

### OPETD-HHDDI interactions promote the decarboxylation of tri-acids

The results in the above section revealed that the co-addition of OPETD and HHDDI in R x 9 (Fig. 5, *D* and *E*) promoted the decarboxylation of tri-acids to form di-acids, whereas R x 5 and R x 6, which included individual OPETD (Fig. 5*D*, *cyan line*) or HHDDI (Fig. 5*D*, *green line*), respectively, led to the accumulation of tri-acids. We speculated that OPETD and HHDDI may form a protein complex that facilitates the decarboxylation process. To test this hypothesis, we first examined the OPETD-HHDDI interaction using Native-PAGE with a controlled loading of 10 μg of each enzyme (Fig. 6*A*). Loading individual control proteins—CHMI, OPETD, and HHDDI—revealed differences in migration and band intensity between OPETD and HHDDI, while CHMI exhibited a migration distance similar to that of OPETD. Co-loading either OPETD or HHDDI with CHMI resulted in migration patterns similar to their respective controls. The combination of CHMI and OPETD showed increased protein band intensity without altering migration, whereas the CHMI-HHDDI mixture exhibited no significant shift relative to the control (Fig. 6*A*). In contrast, the OPETD-HHDDI mixture showed a distinctly altered pattern: a faint band was observed at the HHDDI migration position, accompanied by an intensified band corresponding to the molecular weight of OPETD. This likely indicates the formation of an OPETD-HHDDI complex. We further examined the effects of varying loading ratios on the OPETD-HHDDI complex formation (Fig. S13). When OPETD was in twofold molar excess, the HHDDI band intensity was reduced compared to the control but comparable to that seen in the 1:1 ratio condition. In contrast, when HHDDI was in excess (1:2 ratio), only a slight decrease in HHDDI band intensity was observed. These findings suggest that only a fraction of HHDDI formed a complex with OPETD when OPETD was the limiting component, leaving unbound HHDDI.

To confirm the formation of OPETD-HHDDI complex, we employed SEC technique using an ENrich SEC650 gel filtration column (Biorad). When a 1:1 mixture of OPETD and HHDDI at two concentrations (0.5 and 1.0 mg/ml) was subjected to SEC, the resulting elution chromatograms differed from those of HHDDI and OPETD (Fig. 6*B*) alone, supporting complex formation. The OPETD-HHDDI complex eluted at 14.30 ml, earlier than HHDDI (14.56 ml) and OPETD (14.78 ml), consistent with increased molecular weight. Oligomeric state determination revealed that OPETD is monomeric with a native molecular weight (MW) of 39.22 ± 4.10 kDa (Fig. S5 and Table S3), consistent with its subunit MW of 28.93 kDa (Tables S1 and S3), For HHDDI which has a subunit MW of 24.10 kDa (Tables S1 and S3), exists as a homodimer with a native MW of 45.70 ± 2.12 kDa (Fig. S5 and Table S3). The OPETD-HHDDI complex had a native MW of 53.70 ± 1.20 kDa, consistent with a heterodimer consisting of one OPETD (28.94 kDa) and one HHDDI subunits (24.10 kDa) (Fig. 6*B*). Together, these data confirm the formation of a binary OPETD-HHDDI complex.

To determine whether the OPETD-HHDDI complex was catalytically active, we measured its decarboxylation activity using a synthesized tri-acid substrate (see Experimental procedures) and compared it to the activity of OPETD and HHDDI alone. The complex exhibited substantially higher tri-acid decarboxylation activity—60- and 12-fold greater than OPETD and HHDDI, respectively (Fig. 6*C*). These findings align with R x 9 in the previous section (Fig. 5, *D* and *E*), thus firmly supporting that formation of the OPETD-HHDDI binary complex formation is required for the efficient decarboxylation of tri-acids. Moreover, combining CHMI with the OPETD-HHDDI complex led to a significant enhancement in decarboxylation activity as compared to the complex or CHMI alone (Fig. 6*C*), consistent with the R x 10 in the previous section (Fig. 5, *D* and *E*). Thus, efficient decarboxylation requires, at minimum, the OPETD-HHDDI complex, with CHMI acting as a crucial facilitator of the process.

In *E. coli* W and *E. coli* C, both decarboxylase and isomerase activities are found within a bifunctional enzyme. The X-ray structure of the bifunctional decarboxylase-isomerase enzyme, *Ec*HpcE (equivalent to OPETD and HHDDI in our study), has been reported. The *Ec*HpcE structure revealed a two-lobed architecture comprising N- and C-terminal domains with close similarity in folding (44, 45). Each domain features an incompletely formed barrel fold, and together they form a compact core stabilized by hydrophobic interactions (46). The C-terminal domain harbors a Ca^2+^-coordinated triad of negatively charged glutamate and aspartate residues and assigned as the decarboxylase, while the N-terminal domain is assigned as the isomerase (46).

To gain structural insights into the OPETD and HHDDI complex, we modeled their individual structures using Alphafold3 and compared them to the *Ec*HpcE structure (PDB ID: 1GTT) (Fig. S6). OPETD shares 69% sequence identity with the *Ec*HpcE C-terminal domain, while HHDDI shares 42% identity to the N-terminal domain (Fig. S7). Structural alignment showed that OPETD and HHDDI mimic the folding of the C- and N-terminal domains of *Ec*HpcE, respectively (Fig. 6*D*). Notably, OPETD possesses an extended β-sheet structure, which was not found in the *Ec*HpcE C-terminal domain (Fig. S8). Analysis of the modeled OPETD-HHDDI interface revealed polar contacts and *pi*-*pi* interactions, supporting the formation of a binary complex (Fig. S8). These results suggests that *A. baumannii* TH have evolutionarily separated the isomerase and decarboxylase functions into distinct proteins—HHDDI and OPETD—while still relying on their assembly for optimal catalytic activity.

### The di-acid hydration requires a divalent metal cofactor for OHEDH catalysis

**Figure 7:**
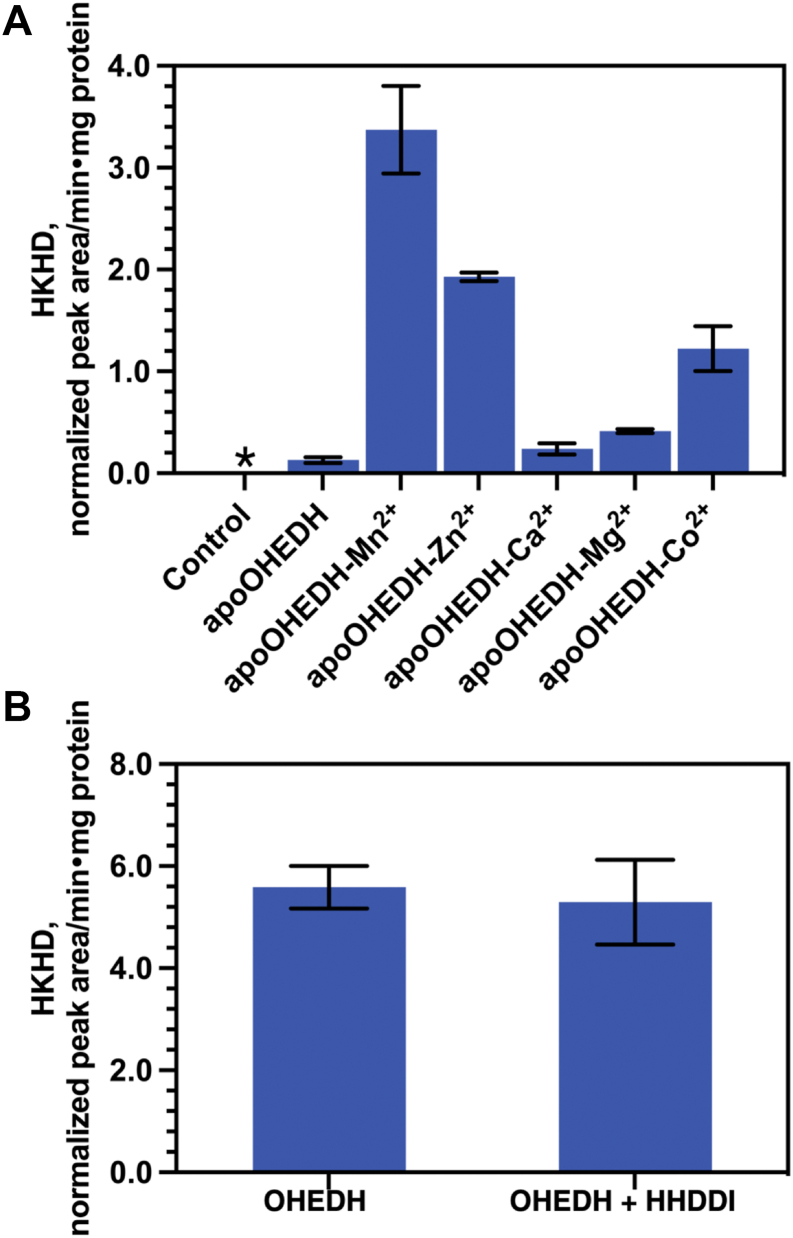
**The effect of Mn^2+^ on OHEDH activity.***A*, the activity of apo-OHEDH reconstituted with various M^2+^ ions. *B*, the Mn^2+^-dependent specific activity of OHEDH in the absence and presence of HHDDI. The *asterisk* indicates no tri-acid hydration activity.

Although hydratases involved in the degradation pathways of aromatic compounds such as phenylpropionate, protocatechuate, and homoprotocatechuate (25, 37, 38) have been investigated, the hydratase from the 4-HPA degradation pathway has not been characterized. Therefore, we investigated the role of metal ions in OHEDH activity. An apo-OHEDH, successfully prepared by treatment with a Chelex 100 chelating resin and verified by the inductively coupled plasma-optical emission spectrometer (ICP-OES) (Fig. S9), was used to reconstitute with various M^2+^ ions, and their enzymatic reactions were tested with the synthesized OHED substrate. The results (Fig. 7*A*) revealed that the OHEDH hydration-specific activity was not detected in apo-OHEDH but showed activity when preincubating apo-OHEDH with M^2+^ ions. The Mn^2+^ ions exhibited the highest activity, followed by Zn^2+^. These findings confirm that a divalent metal ion is required for OHEDH catalysis. Moreover, we investigated whether HHDDI, a putative di-acid isomerase, might facilitate the tautomerization of an enol (HHDD) to a keto form (OHED), the usual substrate for OHEDH. To assess this, OHEDH activity assays were performed in the absence and presence of HHDDI enzyme under Mn^2+^-dependent conditions. The results showed that the specific activities of OHEDH in HKHD production were unchanged, even though HHDDI was included in the reaction (Fig. 7*B*). This suggests that the synthesized OHED substrate predominantly existed in the keto form (OHED) and was already suitable for the OHEDH catalysis.

## Discussion

To our knowledge, this study is the first to fully characterize the cascade reactions and intermediates of the 4-HPA degradation pathway in *A. baumannii* TH. Prior investigations focused only on enzymes at the initial (HPAH and 3,4-DHPAO) and final (HKHDA and SSADH) steps. Here, five putative enzymes in the middle of the pathway, including CHMSD, CHMI, OPETD, HHDDI, and OHEDH, were identified, and their functional roles were comprehensively investigated by *in vitro* one-pot enzymatic cascade reactions. Our findings demonstrated that, apart from synthesizing tri-acids and di-acids, CHMSD can also function to compensate for SSADH activity. Furthermore, the effective decarboxylation of tri-acids requires an OPETD-HHDDI binary complex, with CHMI accelerating the process to produce di-acids, which are then hydrated by OHEDH. This study fills a critical gap in the 4-HPA degradation pathway and lays a foundation for future metabolic engineering and biotechnological applications.

Based on the results obtained from the different multi-setups of *in vitro* enzymatic cascade reactions (Figs. 3 and 5), all pathway intermediates were successfully identified to completely assign all enzymatic reactions of the 4-HPA degradation pathway. Enzyme omission experiments resulted in intermediate accumulation (Figs. 3 and 5), providing insights into the reaction order, similar to findings in the *p*-nitrophenol degradation pathway of *Pseudomonas* sp. (44). In the absence of one or two enzymes in the downstream pathway (Figs. 3*C* and 5*C*), CHMS accumulated at the first 5 min and then decayed. This may suggest that the CHMSD-catalyzed conversion of CHMS into tri-acids (CHM/OPET) is the rate-determining step of the 4-HPA degradation pathway in *A. baumannii*. This contrasts with other reported aromatic-degrading pathways, where the initial double hydroxylation catalyzed by ring hydroxylating dioxygenases in the polycyclic aromatic hydrocarbons (PAHs) degradation pathway (45, 47) and the decarboxylation step in the phthalate degradation pathway of sulfate-reducing bacteria have been identified as rate-limiting steps (48).

The present study demonstrated that, in addition to CHMS, CHMSD can also use SSA—a natural substrate of SSADH—as a substrate (Fig. 4, *A* and *B*), suggesting that SA can be generated by CHMSD catalysis without the addition of SSADH. Although the measured activity of CHMSD toward the SSA substrate (NAD^+^ as a co-substrate) was 20-fold lower than that of SSADH, the dehydrogenase activity level of CHMSD was sufficient to completely convert SSA into SA (Fig. 3, *H* and *I*), implicating that the apparent CHMSD activity is sufficient to support the physiological function in the absence of a canonical SSADH to complete the pathway. The sequence analysis of *A. baumannii* TH CHMSD and CHMSD from other bacterial sources lacking *ssadh* gene in their 4-HPA operon also showed a high sequence identity (74–87%) to the CHMSD studied here (data not shown). These data imply that those CHMSDs may also be able to use SSA as a substrate and compensate for SSADH function to generate SA. Most of the previously reported 4-HPA degradation operons from *P. putida* U, *P. aeruginosa*, *B. xenovoran, Yersinia pestis, S. dublin*, and *K. pneumoniae* lack the *ssadh* gene (10). The findings in this study reveal how these bacterial cells can detoxify SSA, a physiologically toxic substance. Previously, it has been proposed that SSA oxidation in these bacteria could be achieved by SSADH homologues encoded by other operons, *i.e.,* GabD (*gabD*) from γ-aminobutyric acid (GABA) degradation operon and Sad (*yneI*) (43, 49, 50). However, it remained ambiguous how the expressions of GabD and Sad were synchronized with the 4-HPA operon, as they are regulated by different metabolic molecules and mechanisms (49, 50, 51, 52). The expression of the 4-HPA operon is controlled by transcriptional regulation *via* the binding of HpaR to the regulatory element upstream of the *hpaA* gene and the *meta*-cleavage operon. In the presence of 4-HPA, pathway intermediates trigger the release of HpaR and induce gene expression (51, 52), while GabD and Sad expressions are controlled by nitrogen metabolism, to degrade metabolically related compounds, such as putrescine, arginine, ornithine, and GABA (53, 54). Our identification of CHMS conversion as the rate-determining step and SSADH-mimic function of CHMSD imply that CHMSD may regulate the flux of SSA generation to prevent toxic SSA accumulation.

**Figure 8:**
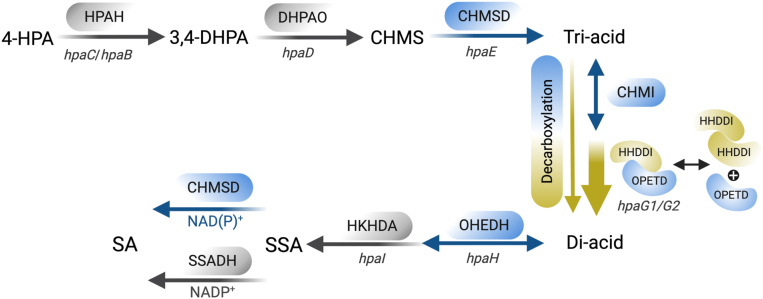
**Reassignment of the cascade enzymes in the 4-HPA degradation pathway in *A. baumannii* TH, which highlights five putative enzymes, including CHMSD, CHMI, OPETD, HHDDI, and OHEDH, located in the middle of the pathway.** CHMSD can compensate for the function of SSADH. The effective decarboxylation activity requires a binary complex of OPETD and HHDDI. Without CHMI, the decarboxylation can proceed, however, the presence of CHMI can accelerate the decarboxylation process by generating a tri-acid isomeric form readily for decarboxylation.

Probing the decarboxylation step—conversion of tri-acids (CHM/OPET) into di-acids (HHDD/OHED)—by different one-pot enzymatic cascade 3,4-DHPA ring-cleavage reactions revealed that effective decarboxylation process occurred when the OPETD-HHDDI complex was formed (Figs. 5*E* and 6*C*). The decarboxylation activity was significantly higher than that of the reaction catalyzed by OPETD alone (Figs. 5*E* and 6*C*). The protein assembly of OPETD and HHDDI to form a binary complex can occur spontaneously without induction by tri-acids (Fig. 6, *A* and *B*, and Table S3). Notably, the highly effective decarboxylation process was achieved when CHMI was also included in the reaction (Fig. 5, *E* and *C*), suggesting that CHMI accelerates the decarboxylation process by catalyzing the isomerization of CHM into OPET, a tri-acid isomeric form ready for decarboxylation (Fig. 8). Since it has been known that the protein-protein interaction could facilitate the direct substrate channeling and improve pathway efficiency (55, 56), the decarboxylation activity enhancement by CHMI may be possibly caused by the transient protein interaction between CHMI and OPETD-HHDDI.

The OPETD and HHDDI in *A.* *baumanii* TH are encoded by separate *hpaG1* and *hpaG2* genes, similar to *B. xenovoran*, *P. aeruginosa and K. pneumoniae* (12, 14, 46, 57). However, their homolog in *E. coli* W (*Ec*HpcE) is found as a single protein of bi-functional decarboxylase/isomerase, consisting of N- and C-terminal domains encoded by the *hpaG* gene (Fig. 5*D*). It is possible that through evolution, the gene was split into *hpaG1* (OPETD) and *hpaG2* (HHDDI); nevertheless, the folding and domain interactions have remained conserved (32). Thus, the interaction between OPETD and HHDDI resembles that of the *Ec*HpcE bi-functional decarboxylase/isomerase (31). It remains ambiguous how these two domains function. Therefore, extensive study should be conducted, including metal ion effects, enzymatic assay, and protein structural complexes with tri-acids. In our case, we speculate that the complex formation of OPETD and HHDDI may increase the structural integrity required for enzyme function.

In conclusion, our results provide a complete identification of all enzymatic functions involved in the degradation of 4-HPA to SA and PYR and offer insights into the reactions catalyzed by five enzymes—CHMSD, CHMI, OPETD, HHDDI, and OHEDH—positioned in the mid-pathway in 4-HPA degradation pathway. We discovered compensatory activity of aldehyde dehydrogenase enzymes within the pathway, as well as the role of substrate isomerization. Additionally, we revealed a protein-protein interaction between OPETD and HHDDI that is essential for efficient decarboxylation (Fig. 8). The OPETD-HHDDI protein complex also reflects the conserved function of these proteins following gene duplication and divergence from the original bifunctional enzyme. Collectively, this study proposes a revised model of the 4-HPA degradation pathway in *A. baumannii* (Fig. 8). The insights gained here may facilitate the future use of 4-HPA degrading enzymes in metabolic engineering and other biotechnological applications.

## Experimental procedures

### Chemicals and reagents

All commercially available chemical reagents were purchased from Sigma-Aldrich, Merck, and Tokyo Chemical Industrial (TCI). Molecular biology reagents were obtained from New England Biolabs (NEB). Plasmid extraction and Gel/PCR mini kits were purchased from Favogen Biotech Corporation. Chromatographic materials were obtained from GE Healthcare. The following buffers were used throughout this study (i) buffer A; 50 mM HEPES, pH 7.0 containing 100 μM PMSF; (ii) buffer B; 50 mM HEPES, pH 7.0; (iii) buffer C; 50 mM HEPES, pH 7.0 containing 50 mM NaCl; (iv) buffer D; 50 mM HEPES, pH 7.0 containing 300 mM NaCl; (v) buffer E; 50 mM HEPES, pH 7.0 containing 15% (w/v) (NH_4_)_2_SO_4_; (vi) buffer F; 50 mM HEPES, pH 7.0 containing 10% (v/v) glycerol; (vii) buffer G; 50 mM HEPES, pH 7.0 containing 150 mM NaCl; (viii) buffer H; 100 mM HEPES, pH 7.0 and (ix) buffer I; 100 mM NH_4_HCO_3_, pH 8.0. Mobile phases for LC included (i) Mobile phase A; 0.1% formic acid in ultrapure water and (ii) Mobile phase B; 0.1% formic acid in acetonitrile.

### Bacterial strains and plasmid construction

*E. coli* XL1-Blue and BL21(DE3) strains, obtained from Novagen, were used for plasmid propagation and protein overexpression, respectively. The recombinant plasmids were constructed using the pET-11a vector (Novagen), harboring genes involved in the 4-HPA degradation pathway of *A. baumannii* TH, including genes *c*_*1*_ (C1-flavin reductase), *dhpao* (3,4-DHPAO), *chmsd* (CHMSD), *chmi* (CHMI), *opted* (OPETD), *hhddi* (HHDDI), and *ohedh* (OHEDH). Genes were amplified using Phusion High-Fidelity DNA polymerase and specific primers (Table S4), with genomic DNA of *A.* *buamannii* TH as a template (10). Constructs encoding 3,4-DHPAO, SSADH, and HKHDA were obtained from previous studies (10, 21, 22, 25).

### Expression and purification of enzymes

All recombinant enzymes were overexpressed in *E. coli* BL21(DE3) using ZYP-5052 rich medium at 25 °C with 100 μg/ml ampicillin. Unless stated otherwise, the purification procedure of all enzymes was carried out at 4 °C. The purification of C1-flavin reductase, 3,4-DHPAO, SSADH, and HKHDA followed previously published protocols (10, 21, 22, 25), with slight modifications (Table S1). For CHMSD, CHMI, OPETD, HHDDI, and OHEDH, cell pellets were resuspended in buffer A and lysed *via* ultrasonication. Cell debris was removed by centrifugation, and the supernatant was treated with PEI to eliminate nucleic acids and nucleic acid-binding proteins. Following centrifugation, (NH_4_)_2_SO_4_ precipitation was performed. The resuspended protein pellet was dialyzed overnight against buffer B, and then was loaded onto a DEAE-Sepharose column pre-equilibrated with buffer C. After washing with buffer C, the protein was eluted using a linear NaCl gradient from buffer C to D. Fractions containing the target enzyme were pooled, concentrated, and precipitated using 50% (v/v) saturated (NH_4_)_2_SO_4._ The protein was resuspended in buffer B and applied to a Phenyl-Sepharose column pre-equilibrated with buffer E. Elution was performed using a decreasing (NH_4_)_2_SO_4_ gradient from buffers E to B. Enzyme-containing fractions were pooled desalted using a Sephadex G-25 column pre-equilibrated with buffer F. The enzyme concentration was determined based on the molar absorption coefficient at absorbance 280 nm (ϵ_280_), calculated from amino acid sequences using the online tool on the ProtParam program of ExPaSy Proteomics Server (http://web.expasy.org/protparam/). Protein concentration was determined using the Bradford assay (58), and the total amount of purified protein in units of mg protein/g cell paste and mg protein/l media culture was then calculated (Table S1). The purity and subunit MW of each enzyme were analyzed by 15% (w/v) SDS-PAGE (Table S1 and Fig. S1). All the purified enzymes were stored at −80 °C until use.

### Determination of native molecular weight of enzymes

The oligomeric state of purified proteins was determined using an ENrich SEC650 (10 × 300 mm) gel filtration column equipped with an NGC Scout Plus FPLC system (BioRad). The column was pre-equilibrated in buffer G at a flow rate of 0.5 ml/min and 4 °C. Protein standards (0.5–1.0 mg/ml) of known MW were prepared and subjected into the column. The protein was eluted with isocratic flow of buffer G while monitoring at absorbance 280 nm. The calibration curve of the protein standards was plotted by linear regression between the gel-phase distribution coefficient (Kva) and the logarithm of the protein MW (Fig. S5). The purified enzyme (0.5–1.0 mg/ml) was then injected into the column. The native MW of each enzyme was determined from the calibration curve plot. The oligomeric state of the purified enzyme was estimated based on the theoretical subunit MW (Tables S1 and S3).

### Identification of intermediates of 4-HPA degradation pathway

To probe the intermediates formed during the conversion of 3,4-DHPA to the end of the cascade reaction, all the enzymes (10 μM each), including activated 3,4-DHPAO, CHMSD, CHMI, OPETD, HHDDI, OHEDH, and HKHDA, were added to the reaction containing 2 mM 3,4-DHPA, 5 mM NAD^+^, and 1 mM MgCl_2_ in a freshly prepared buffer I. The C1 flavin reductase (20 μM)-catalyzed NAD^+^/NADH regenerating system was applied to replenish NAD^+^ cofactor, and catalase (3 mg/ml) was added to scavenge the reactive oxygen species. The protocol for 3,4-DHPAO activation was described previously (21). The reaction was aliquoted at different time intervals and then quenched with 0.5 M HCl containing 200 μM pimelic acid as an internal standard (ISTD). The quenched samples were centrifuged at 13,800*g* and 4 °C for 10 min to separate the denatured protein, which was subsequently collected and filtered through 0.22 μm nylon filter membrane. Two microliters of the filtrate were injected into a LC system coupled with high-resolution MS.

The LC condition was optimized for separating substrates and product intermediates using ZORBAX Sb-Aq C18 column (5 μm, 4.6 × 150 mm) (Agilent Technologies). The column was equilibrated with mobile phase A with a flow rate of 0.5 ml/min for 30 min before analysis. After the reaction sample was injected into the column, the column was washed with mobile phase A for 8 min, then subjected to an increasing gradient of 0 to 15% of mobile phase B for 9 min, and maintained at 15% of mobile phase B for 3 min. The column condition was subsequently returned to the initial condition with mobile phase A for 5 min and held at this condition for 5 min. Thus, the total running time was 30 min, and the column was pre-equilibrated with mobile phase A at least 5 min before injecting the next sample. Each compound was eluted out from the column and detected directly by a MS using negative ionization mode. All product intermediates were identified based on their predicted mass-to-charge ratios (m/z) at specific retention times compared with the reaction without those enzymes (control) (Table S2).

### Investigation of CHMSD and SSADH activities on the oxidation of CHMS and SSA substrates

To investigate the ability of CHMSD to use SSA, a native substrate of SSADH, the CHMSD activity assay was carried out in a 1 ml reaction containing 1 mM SSA and 1 mM NAD(P)^+^ in buffer H. The reaction was initiated by adding CHMSD (0.005–1 μM). The NAD(P)H production was monitored for the absorbance increase at 340 nm. The specific activity of CHMSD was determined based on a molar absorption coefficient (ϵ_340_) of 6.22 mM^−1^ cm^−1^ of NAD(PH) (59) and compared to that of the reaction using SSADH.

Similarly, the activity of SSADH using CHMS, a native substrate of CHMSD, as a substrate was also performed. First, CHMS was synthesized from the 3,4-DHPA aromatic ring cleavage reaction containing 4 mM 3,4-DHPA and 20 μM activated 3,4-DHPAO in buffer H. The CHMS formed was identified by its yellow color with a maximum absorption at 380 nm. The 3,4-DHPAO was then removed from the mixture by ultrafiltration. The CHMS concentration was calculated using a molar absorption coefficient (ϵ_380_) of 32.23 mM^−1^ cm^−1^ (21). To perform the SSADH assay, the reaction contained 25 μM CHMS and 1 mM NAD(P)^+^ in buffer H and was initiated by adding SSADH (0.002–0.010 μM). The CHMS oxidation was monitored by measuring the depletion of absorbance at 380 nm. The specific activity of SSADH was determined based on the ϵ_380_ value of CHMS and compared to that of the reaction using CHMSD.

### One-pot enzymatic synthesis of di- and tri-acid under auxiliary reactions

A one-pot cascade reaction was developed to synthesize the tri-acid, CHM/OPET, from 3,4-DHPA (Fig. S10). Two auxiliary systems were included: an NAD^+^ regeneration system driven by C1-reductase and the reactive oxygen species scavenging system catalyzed by catalase. The reaction consisted of 10 μM of each enzyme (activated 3,4-DHPAO and CHMSD), 0.5 mM NAD^+^, 0.5 mM MgCl_2,_ 20 μM C1-reductase, and 3 mg/ml catalase in buffer I. For the synthesis of di-acid, HHDD/OHED, the cascade reaction was carried out using the same setup, with the addition of CHMI, OPETD, and HHDDI enzymes at final concentration of 10 μM each (Fig. S10).

For large-scale synthesis of tri-acid (CHM/OPET) compounds, the reaction contained 20 μM activated 3,4-DHPAO, 20 μM C1-reductase, 50 μM CHMSD, 0.5 mM NAD^+^, 0.5 mM MgCl_2_, 3 mg/ml catalase, and 20 mM 3,4-DHPA in buffer I. For HHDD/OHED synthesis, the procedure was modified by adding 50 μM each of CHMI, OPETD, and HHDDI. The reactions were incubated for 3 h at ambient temperature. To terminate the reaction, 6 M HCl was added to adjust the pH to 3.0, precipitating the proteins. The precipitates were removed by centrifugation at 18,514*g* and 4 °C, for 10 min. The supernatant containing product intermediates was partially purified by liquid-liquid extraction technique using a 1:2 volume ratio of ethyl acetate. The upper phase was separated and evaporated using a Buchi Rotavapor R-100 (India). The dried extract was re-dissolved in 50% (v/v) methanol in distilled water and stored at −20 °C until use. The UV-visible absorption and mass spectra of partially purified di-and tri-acid compounds were characterized by LC-DAD/MS (Figs. S10 and S11).

### Investigation of tri-acid decarboxylation reaction

Enzymatic decarboxylation of tri-acid was performed in buffer H consisting of partially purified tri-acid, 10 μM of each enzyme (CHMI, OPETD and HHDDI), and 0.5 mM MgCl_2_. The reaction was then quenched at different time intervals with 0.5 M HCl containing 200 μM ISTD. The quenched samples were processed as described above, and the predicted m/z of the di-acid were analyzed by LC-MS/MS.

To determine the enzyme requirements for CHM decarboxylation into OHED, reactions were run with the omission of CHMI, OPETD, or HHDDI individually, using identical tri-acid preparations. The di-acid product was confirmed by m/z and compared with that obtained from the reaction without enzymes (control). The time-course di-acid production was plotted based on ISTD-normalized peak area, and decarboxylation rates were calculated as normalized peak area/min/mg of protein.

### Investigation of protein-protein interaction monitored by size-exclusion techniques

To investigate whether interactions among CHMI, OPETD, and HHDDI influence tri-acid decarboxylation, Native-PAGE and FPLC were employed. 10 μg of each protein was incubated in buffer B on ice for 30 min and then loaded onto a Native-PAGE gel. To confirm the protein complex formation of CHMI, OPETD, and HHDDI enzymes, the FPLC was operated under the same conditions described earlier. The mixture of OPETD and HHDDI was incubated in buffer G on ice with a 1:1 M ratio. The protein mixture solution was filtered through a 0.22 μm cellulose filter membrane prior to applying to the FPLC. The proteins in the eluted fractions were analyzed for their native MWs and for subunit MWs by SDS-PAGE.

### Apo-enzyme preparation of OHEDH and metal content analysis

All materials were pre-treated with 2% (v/v) nitric acid in ultrapure water to remove trace metals. Purified OHEDH was incubated with Chelex 100 resin (Merck KGaA) overnight at 4 °C to strip metal ions. The resin was removed by centrifuged at 13,800*g* and 4 °C for 30 min, yielding apo-OHEDH. To assess metal ion binding, apo-OHEDH was incubated with a 10-fold molar excess of different types of divalent metal chloride, including MnCl_2_, MgCl_2_, ZnCl_2_, and CaCl_2_, overnight at 4 °C. Excess metals were removed using Sephadex gel filtration using a PD-10 column pre-equilibrated with buffer B. The protein samples, including the purified, apo-enzyme, and metal-substituted apo-enzyme, at a concentration of 40 μM were subjected into ICP-OES to measure the metal contents. All emission signals were subtracted from buffer B (control). The calibration curve plots of metal were obtained from varying metal concentrations in a range of 0 to 4 ppm in 2% (v/v) nitric acid, and emission intensity was analyzed under the same conditions as the enzyme. The metal contents of OHEDH samples were reported as a metal mole ratio per enzyme subunit.

### Metal dependent-hydration activity of OHEDH

To assess metal effects on OHEDH activity, apo-OHEDH was reconstituted by incubating with a 200-fold molar excess of various metal chloride in metal-free buffer J for 30 min at 25 °C. The OHEDH activity was performed using a partially purified OHED substrate and OHEDH (5–100 nM). Reactions were aliquoted at different time points and then quenched with 0.5 M HCl containing 200 μM ISTD. The quenched samples were processed as described above, and the predicted m/z of the hydration product HKHD was analyzed by LC-MS/MS. The specific activity of OHEDH was determined based on the HKHD formation rate measured. Hydratase activity was also evaluated in the presence of 100 nM HHDDI to assess whether HHDD isomerization influences OHEDH-mediated hydration.

## Data availability

The data that support the findings of this study are available from the corresponding author upon reasonable request.

## Supporting information

This article contains supporting information.

## Conflicts of interest

The authors declare no commercial or financial conflict of interest with the contents of this article.

## Supporting information

Suppoting Information
