# Supplementary material for: Discovery and biochemical characterization of enzymes completing the 4-hydroxyphenylacetate pathway in Acinetobacter baumannii TH
Source: J Biol Chem. 2025 Nov 5;301(12):110917. doi: 10.1016/j.jbc.2025.110917 (PMC12718150; doi:10.1016/j.jbc.2025.110917)
Supplement: Suppoting Information [file mmc1.docx]

**Supporting Information**

**Discovery and biochemical characterization of enzymes completing the 4-hydroxyphenylacetate pathway in *Acinetobacter baumannii* TH**

**Wachirawit Chinantuya^1^, Kittipop Kungchuai^2,3^, Pimchai Chaiyen^2^, Somchart Maenpuen^3*^, and Ruchanok Tinikul^1*^**

^1^Department of Biochemistry and Center for Excellence in Protein and Enzyme Technology, Faculty of Science, Mahidol University, Bangkok 14000, Thailand.

^2^School of Biomolecular Science and Engineering, Vidyasirimedhi Institute of Science and Technology (VISTEC), Wangchan Valley, Rayong 21210, Thailand.

^3^Department of Biochemistry, Faculty of Science, Burapha University, Chonburi 20131, Thailand.

Running title: The complete 4-HPA degradation pathway in *A. baumannii* TH.

^*^To whom correspondence should be addressed: Ruchanok Tinikul, Department of Biochemistry and Center for Excellence in Protein and Enzyme Technology, Faculty of Science, Mahidol University, Bangkok 10400, Thailand;

E-mail: [ruchanok.tin@mahidol.ac.th](mailto:ruchanok.tin@mahidol.edu); Tel. +66-2201-5607, Fax. +66-2201-5843.

and Somchart Maenpuen, Department of Biochemistry, Faculty of Science, Burapha University, 169 Long-Hard Bangsaen Road, Chonburi 20131, Thailand;

Email: [somchart@buu.ac.th](mailto:somchart@buu.ac.th); Tel. (+66)-3810-3058 (ext. 29); Fax. (+66)-3839-3495.

**List of supporting materials**

**1. Supporting tables: 4**

**2. Supporting figures and legends: 13**

**Supporting tables**

**Table S1. Summary of purification protocols and molar absorption coefficient for enzymes in this study.**

| **Enzymes** | **Purification steps** | | **Total amount of purified protein** | | **Subunit MW (kDa)/pI^a^** | **ɛ_280_**  **(mM^-1^cm^-1^)^a^** | **Purification protocol** |
| --- | --- | --- | --- | --- | --- | --- | --- |
|  | **%**  **(w/v)**  **PEI** | **%**  **(w/v)**  **(NH_4_)_2_SO_4_** | **mg protein/**  **g cell paste** | **mg protein/**  **l media culture** |  |  |  |
| C1 reductase | 1.0 | 40-80 | 3.82 | 51.92 | 35.73/6.02 | 12.80^b^ | Modified from (21) |
| 3,4-DHPAO | 0.5 | 20-40 | 4.27 | 63.46 | 32.37/5.53 | 53.78 | Modified from (13) |
| CHMSD | 0.2 | 0-20 | 3.92 | 52.31 | 53.01/6.05 | 49.77 | This study |
| CHMI | 1.0 | 0-40 | 5.30 | 61.20 | 14.80/6.06 | 4.47 | This study |
| OPETD | 0.2 | 30-50 | 7.62 | 91.88 | 28.93/4.99 | 37.49 | This study |
| HHDDI | 0.2 | 40-50 | 5.83 | 80.77 | 24.10/5.32 | 19.04 | This study |
| OHEDH | 0.1 | 10-30 | 11.03 | 118.80 | 29.41/5.39 | 19.94 | This study |
| HKHDA | 0.5 | 20-40 | 7.53 | 92.69 | 28.75/4.95 | 30.04 | Modified from (24) |
| SSADH | 0.5 | 0-40 | 6.75 | 65.81 | 52.31/5.62 | 49.64 | Modified from (22) |

^a^The theoretical subunit MW, pI and molar absorption coefficient were calculated based on the deduced amino acids using the ProtParam program on the ExPaSy Proteomics Server (https://web.expasy.org/protparam/). ^b^The concentration of C1 reductase bound FAD was measured based on the molar absorption coefficient at 458 nm.

**Table S2. Chemical information of product intermediates detected in 4-HPA degradation pathway.**

| **Chemical name** | **Abbreviation** | **R_t_ (min)** | **Wavelength**  **(nm)** | **m/z of**  **Extract ion^a^** |
| --- | --- | --- | --- | --- |
| 3,4-Dihydroxyphenylacetate | 3,4-DHPA | 18.281 | 280 | 167 |
| 5-Carboxymethyl-2-hydroxymuconate semialdehyde | CHMS | 19.101 | 310 | 199 |
| 5-Carboxymethyl-2-hydroxy-muconate (CHM)/  5-oxo-pent-3-ene-1,2,5-tricarboxylate (OPET) | Tri-acid | 18.721 | 310 | 215 |
| 2-Hydroxy-hept-2,4-diene-1,7-dioate (HHDD)/  2-Oxo-hept-3-ene-1,7-dioate (OHED) | Di-acid | 19.673 | 280 | 171 |
| 4-Hydroxy-2-keto-heptane-1,7-dio | HKHD | 10.85 | - | 189 |
| Pyruvate | PYR | 5.161 | - | 87, 105^b^ |
| Succinic semialdehyde | SSA | 5.820 | - | 101 |
| Succinic acid | SA | 7.043 | - | 117 |
| Mixed coenzyme | NADPH | 9.028 | 254 | 743 |
|  | NADH | 14.330 | 254 | 662 |
| Flavin mononucleotide | FMN | 20.659 | 254 | 455 |
| Pimelic acid (ISTD) | PA | 21.102 | - | 159 |

^a^The m/z of extract ions were detected in a negative mode of mass spectrometer. ^b^The m/z of a water adduct of PYR.

**Table S3. The oligomeric state of purified protein and proteins complex in 4-HPA degradation pathway.**

| **Enzyme** | **Elution volume (ml)** | **Kva** | **logMw** | **Native molecular weight**  **(MW)** | **Subunit (kDa)** | **Oligomeric state** |
| --- | --- | --- | --- | --- | --- | --- |
| 1.0 mg/ml of CHMSD | 12.76 | 0.16 | 2.19 | 155.09 ± 2.08 | 53.01 | homotrimer |
| 1.0 mg/ml of CHMI | 15.00 | 0.34 | 1.51 | 32.61 ± 1.70 | 15.00 | homodimer |
| 1.0 mg/ml of OPETD | 14.90 | 0.33 | 1.54 | 39.22± 4.10 | 28.94 | monomer |
| 1.0 mg/ml of HHDDI | 14.56 | 0.30 | 1.65 | 45.70 ± 2.12 | 24.10 | homodimer |
| 1.0 mg/ml of OHEDH | 11.78 | 0.08 | 2.49 | 313.27 ± 5.69 | 29.80 | homodecamer |
| 1.0 mg/ml of combined OPETD and HHDDI | 14.27 | 0.28 | 1.73 | 53.50± 0.70 | 53.04 (24.10+28.94) | heterodimer of HHDDI and OPETD |

**Table S4.** **Primer sequences for amplification of genes involved in 4-HPA degradation pathway of *A. baumannii.***

| **Gene** | **Primer** | **Nucleotide Sequence (5’→3’)** |
| --- | --- | --- |
| *hpaF/chmi* | Forward | GGAATTCCATATGCCACACTTTATTGCTGAGTAC |
|  | Reverse | CGCGGATCCTCAAGCCTGATTTTTTAAAAACTGATGG |
| *hpaG2/opetd* | Forward | GGAATTCCATATGAAGACAGCTAAAATTAAATATG |
|  | Reverse | CGCGGATCCTTATTTCACAGTATTAAAAAATTCAG |
| *hpaG1/hhddi* | Forward | GGAATTCCATATGGATCACTCAAAAGAGATC |
|  | Reverse | CGCGGATCCCTATGCTCCCTGTTCCTGAACAG |
| *hpaH/ohedh* | Forward | CGCGGATCCATGTTAGATAAAGCCAAAATTCAAG |
|  | Reverse | CGCGGATCCTTATACAAAGCGAAAAGCAATTG |

**Supporting figures**

**
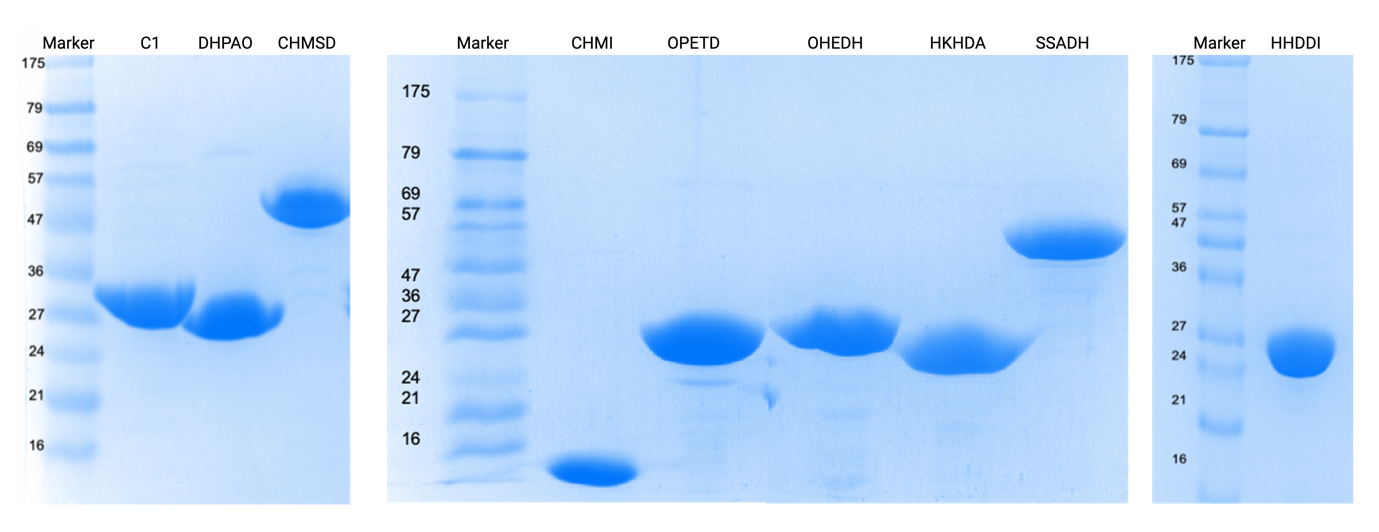
**

**Figure S1. SDS-PAGE analysis of purified enzymes involved in 4-HPA degradation pathway of *A. baumannii*.** A 20 µg of purified enzymes was loaded on a 15% SDS-PAGE. Lane 1, protein marker; Lane 2, C1-reductase; Lane 3, 3,4-DHPAO; Lane 4, CHMSD; Lane 5, protein marker; Lane 6, CHMI; Lane 7, OPETD; Lane 8, OHEDH; Lane 9, HKHDA; Lane 10, SSADH; Lane 11, protein marker; and Lane 12, HHDDI.

**
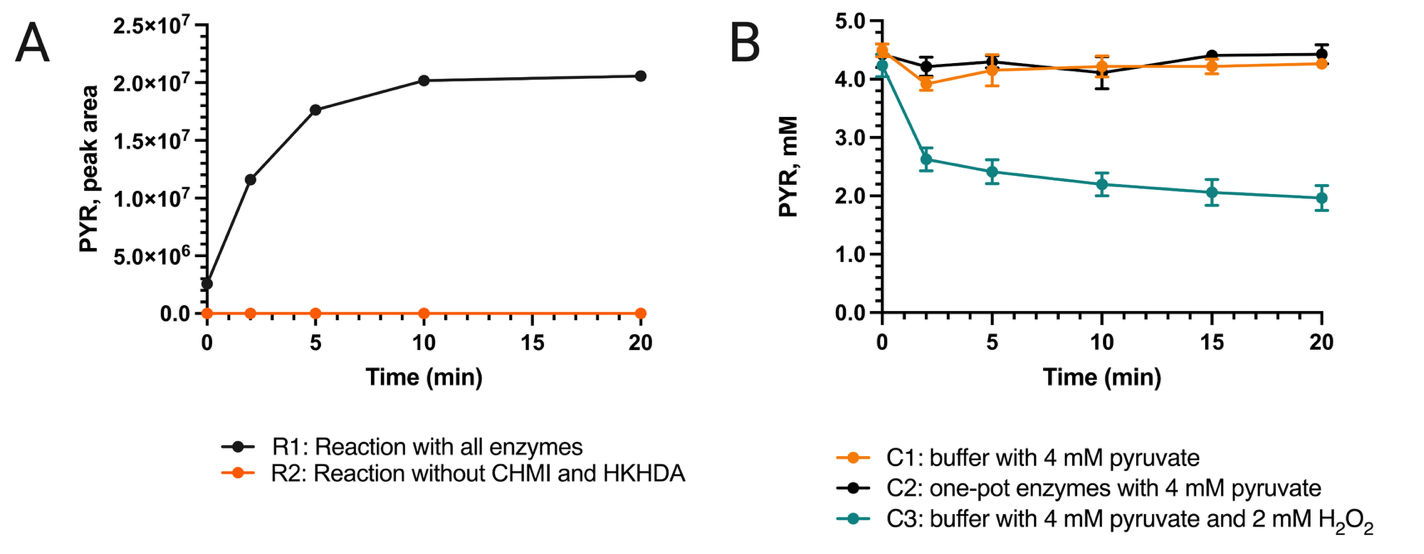
**

**Figure S2.** **The investigation of pyruvate formation and pyruvate stability under different conditions.** (A) Pyruvate formation in a one-pot cascade enzymatic reaction of 3,4-DHPA without flavin reductase-catalyzed NAD^+^/NADH regenerating system, R1 (black line), reaction with all enzymes (3,4-DHPAO, CHMSD, CHMI, OPETD, HHDDI, OHEDH, and HKHDA) and R2 (orange line), reaction without CHMI and HKHDA. (B) The effect of H_2_O_2_ on pyruvate stability, the reaction C1 (yellow line), pyruvate was mixed with all pathway enzymes (3,4-DHPAO, CHMSD, CHMI, OPETD, HHDDI, OHEDH, HKHDA, and SSADH) and 4 mM of pyruvate without the addition of 3,4-DHPA (a starting substrate). The reaction C2 (black line), pyruvate alone (control reaction) and reaction C3 (cyan line), pyruvate was mixed with 2 mM H_2_O_2_.

**
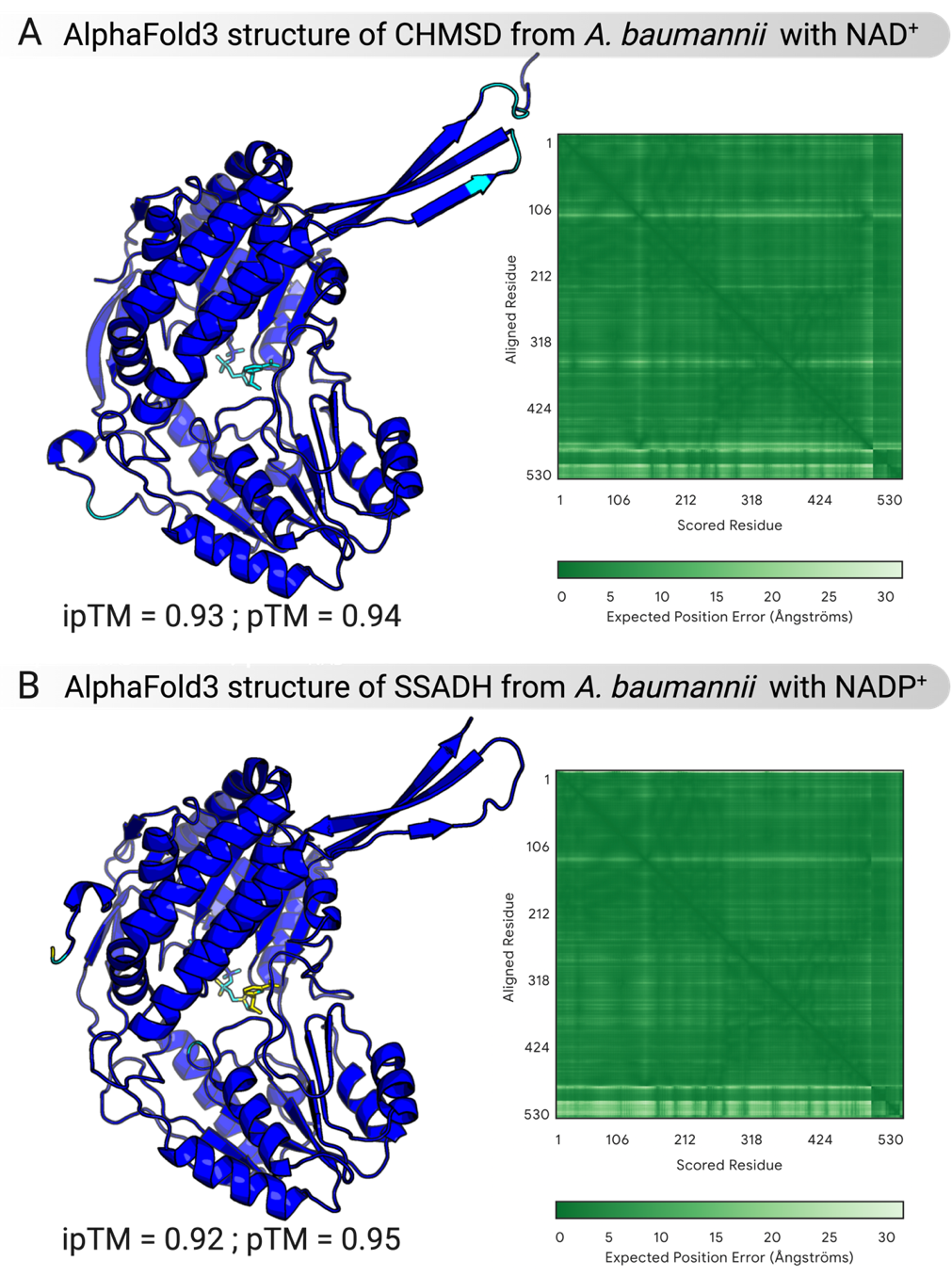
**

**Figure S3. Alphafold3-predicted structures and representative predicted aligned error (PAE) plots for *Ab*CHMSD and *Ab*SSADH.** (A) The *Ab*CHMSD model with incorporated NAD^+^, achieved high confidence scores for the predicted interface (ipTM) and the overall complex structure (pTM), with values of 0.93 and 0.94, respectively. (B) The *Ab*SSADH mode with incorporated NADP^+^, achieved high confidence scores for the predicted interface (ipTM) and the overall complex structure (pTM), with values of 0.92 and 0.95, respectively. The coloring structures are defined by the estimated confidence pLDDT value, the blue color indicates a high-confidence structure, while red color indicates a low-confidence structure. The PAE score reflects the expected positional error between the predicted and actual structures, darker green indicates a low PAE, suggesting a high reliability of the relative positions of the residues, while lighter colors indicate lower confidence.


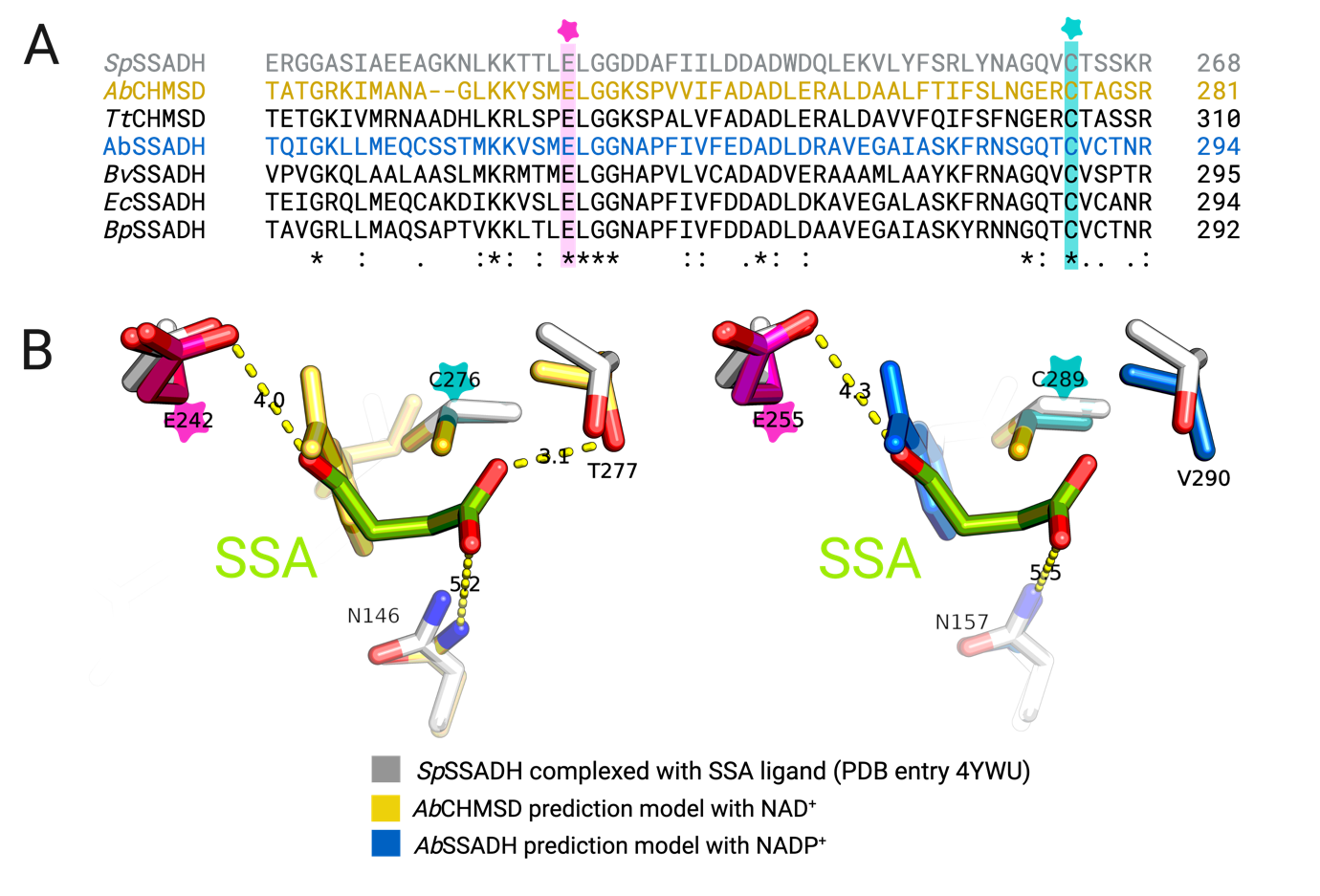
**Figure S4.** **Sequence alignment and the active site models of *Ab*CHMSD and *Ab*SSADH.** (A) The sequence alignment of *Ab*CHMSD and *Ab*SSADH with homologous enzymes, including *Sp*SSADH from complex from *Streptococcus pyogenes* MGAS1882, *Tt*CHMSD from *Thermus thermophilus* HB8, *Bv*SSADH from *Burkholderia vietnamiensis*, *Ec*SSADH from *E. coli* K-12 and *Bp*SSADH from *Burkholderia pseudomallei* 1710b, shows conserved catalytic cysteine (pink highlight) and glutamate residue (cyan highlight) essential for enzyme catalysis. (B) The active site of model structures of *Ab*CHMSD and *Ab*SSADH generated by Alphafold3, superimposed with *Sp*SSADH-SSA complex (PDB entry 4YWU) and visualized using PyMOL.


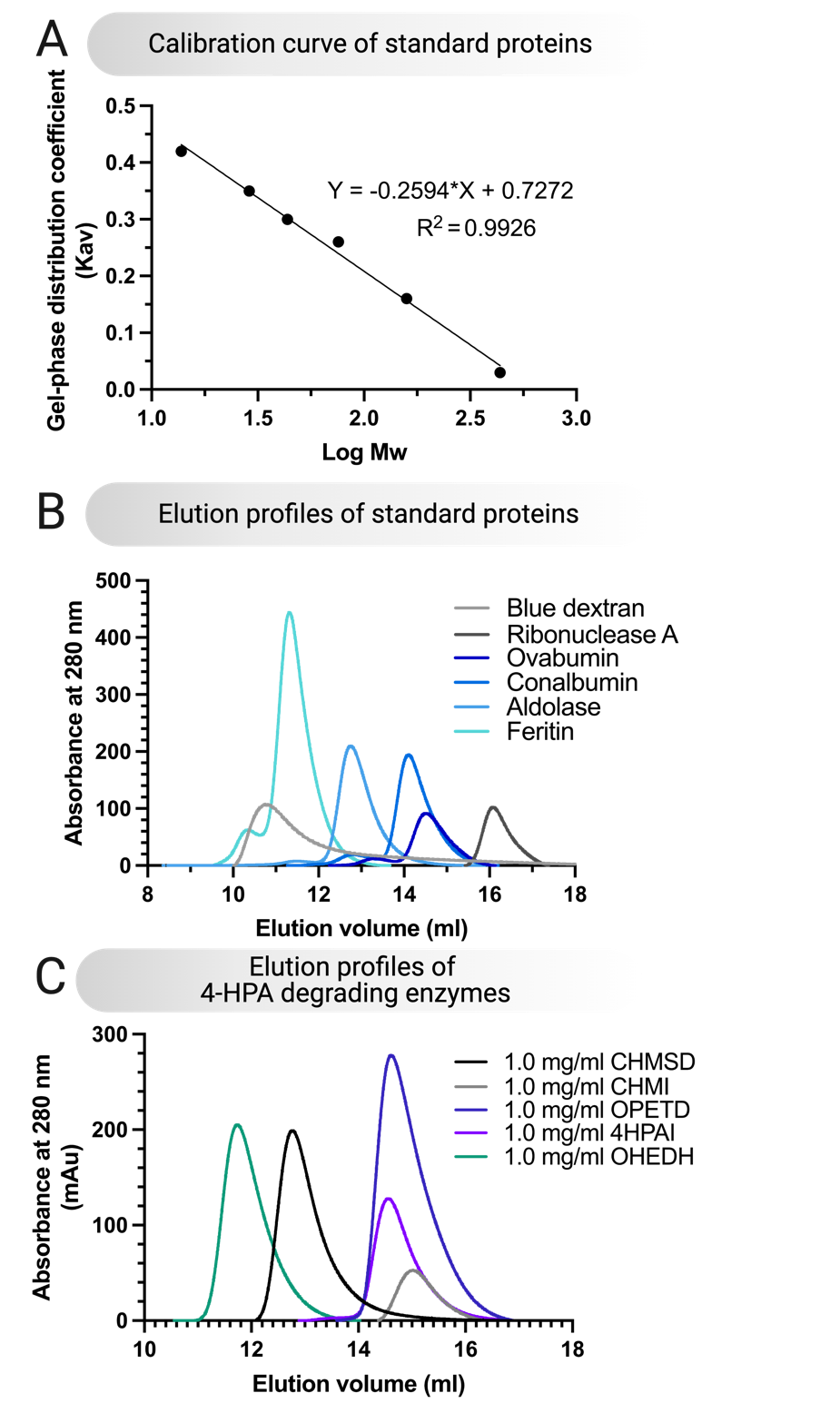


**Figure S5. The calibration curve plot and FPLC chromatograms of enzymes involved in 4-HPA degradation pathway**. (A) The calibration curve plot of standard proteins, which include ferritin (440 kDa), aldolase (158 kDa), conalbumin (75 kDa), ovalbumin (44 kDa), carbonic anhydrase (29 kDa) and ribonuclease A (13.7 kDa). Blue dextran (2000 kDa) was used to measure a void volume. (B) The elution profiles of standard proteins. (C) The elution profiles of enzymes in 4-HPA degradation pathway of *A. baumannii*, which include CHMSD, CHMI, OPETD, HHDDI and OHEDH and their native MWs were determined accordingly **(Table S3)**.

**
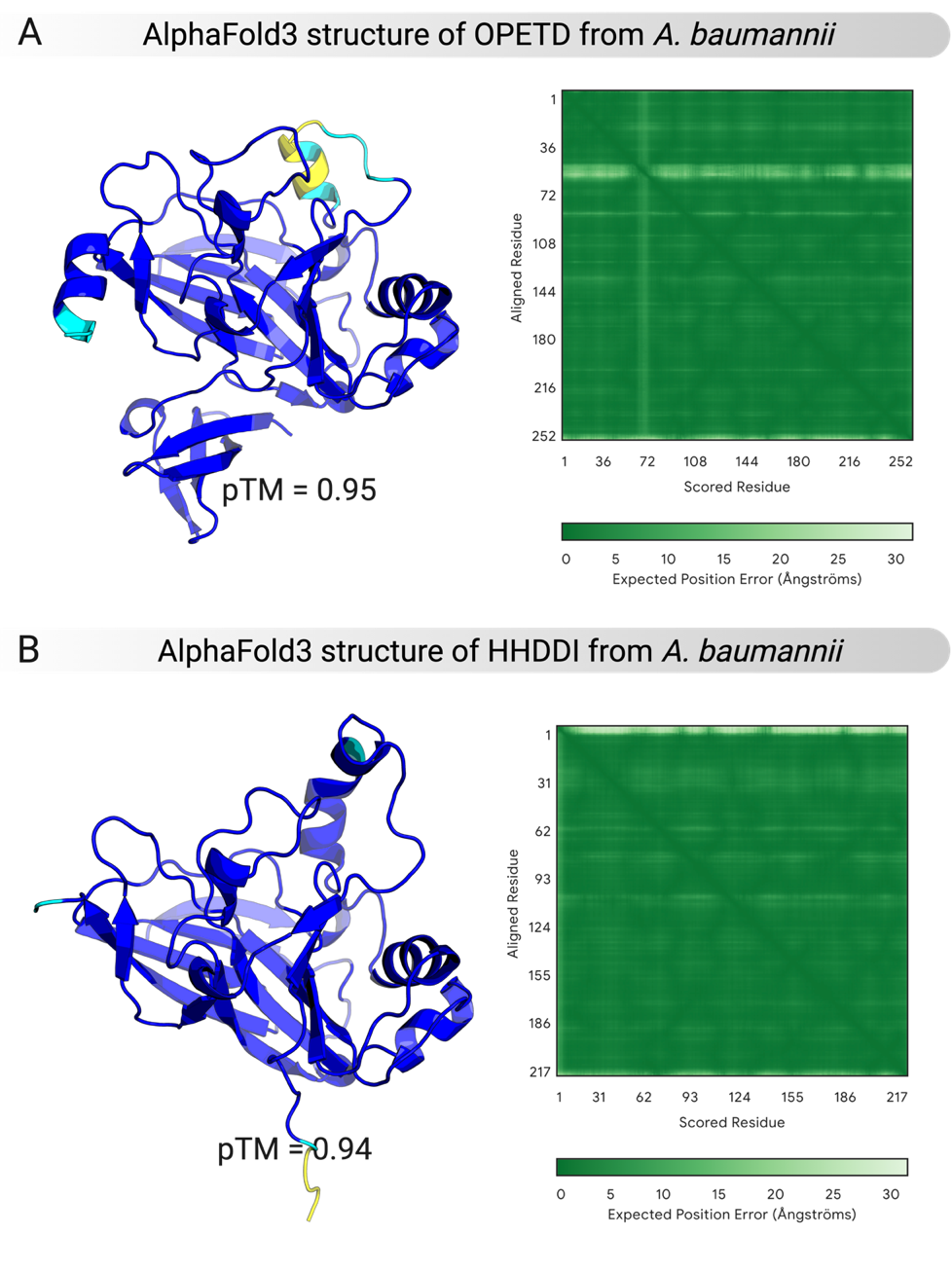
**

**Figure S6. Alphafold3-predicted structures and representative predicted aligned error (PAE) plots for *Ab*OPETD and *Ab*HHDDI are presented below.** (A) The *Ab*OPETD model achieved high confidence scores for the overall complex structure (pTM), with values of 0.95. (B) The *Ab*HHDDI model achieved high confidence scores for the overall complex structure (pTM), with values of 0.94. The coloring structures are defined by the estimated confidence pLDDT value, the blue color indicates a high-confidence structure, while red color indicates a low-confidence structure. The PAE score reflects the expected positional error between the predicted and actual structures, darker green indicates a low PAE, suggesting a high reliability of the relative positions of the residues, while lighter colors indicate lower confidence.


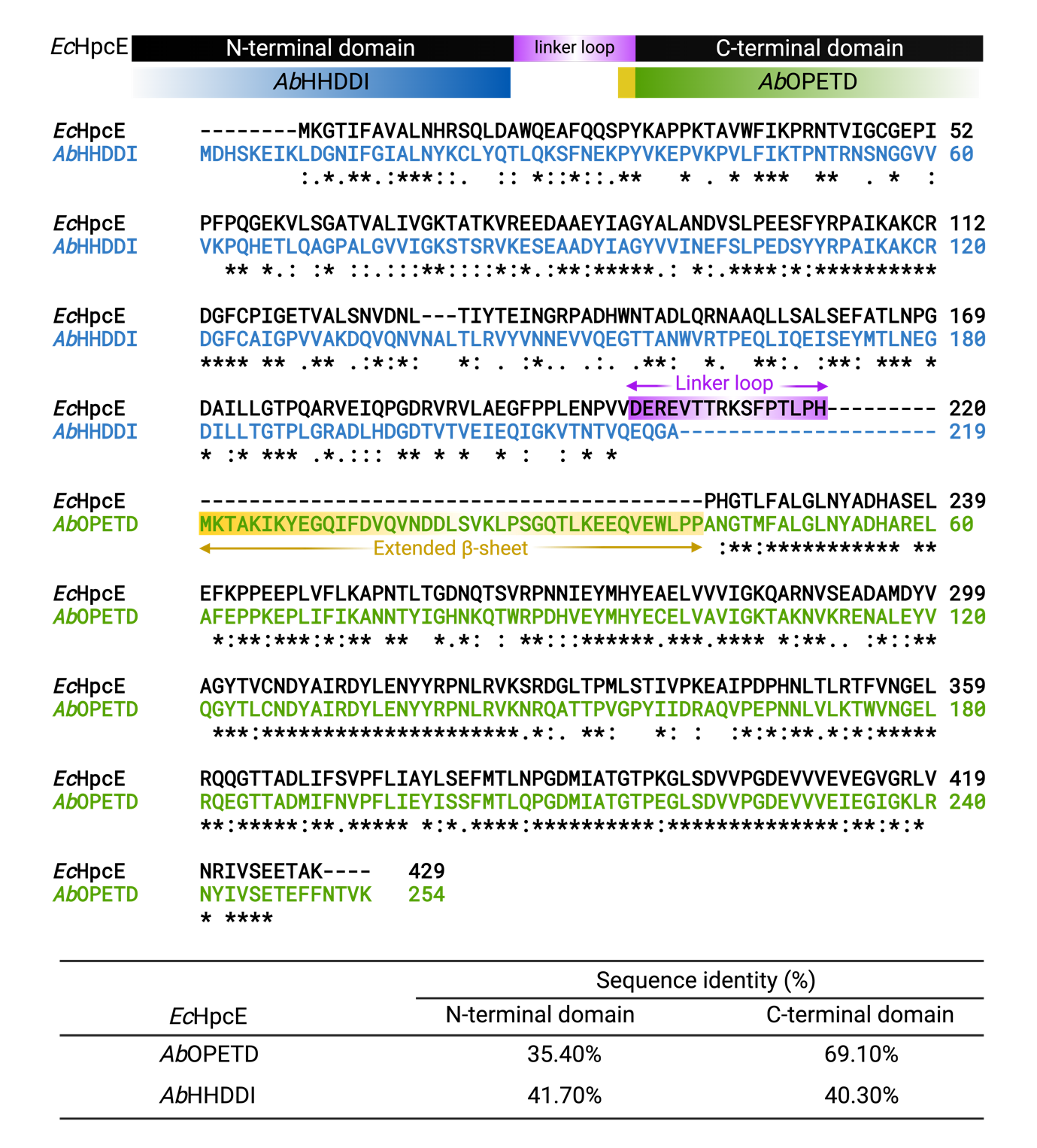


**Figure S7**. The sequence resembles HHDDI and OPETD toward the N- and C-terminal domain sequences of *Ec*HpcE bifunctional enzyme (UniProt entry Q46978) and percent identity matrix analyzed by UniProt web-based tool.

**
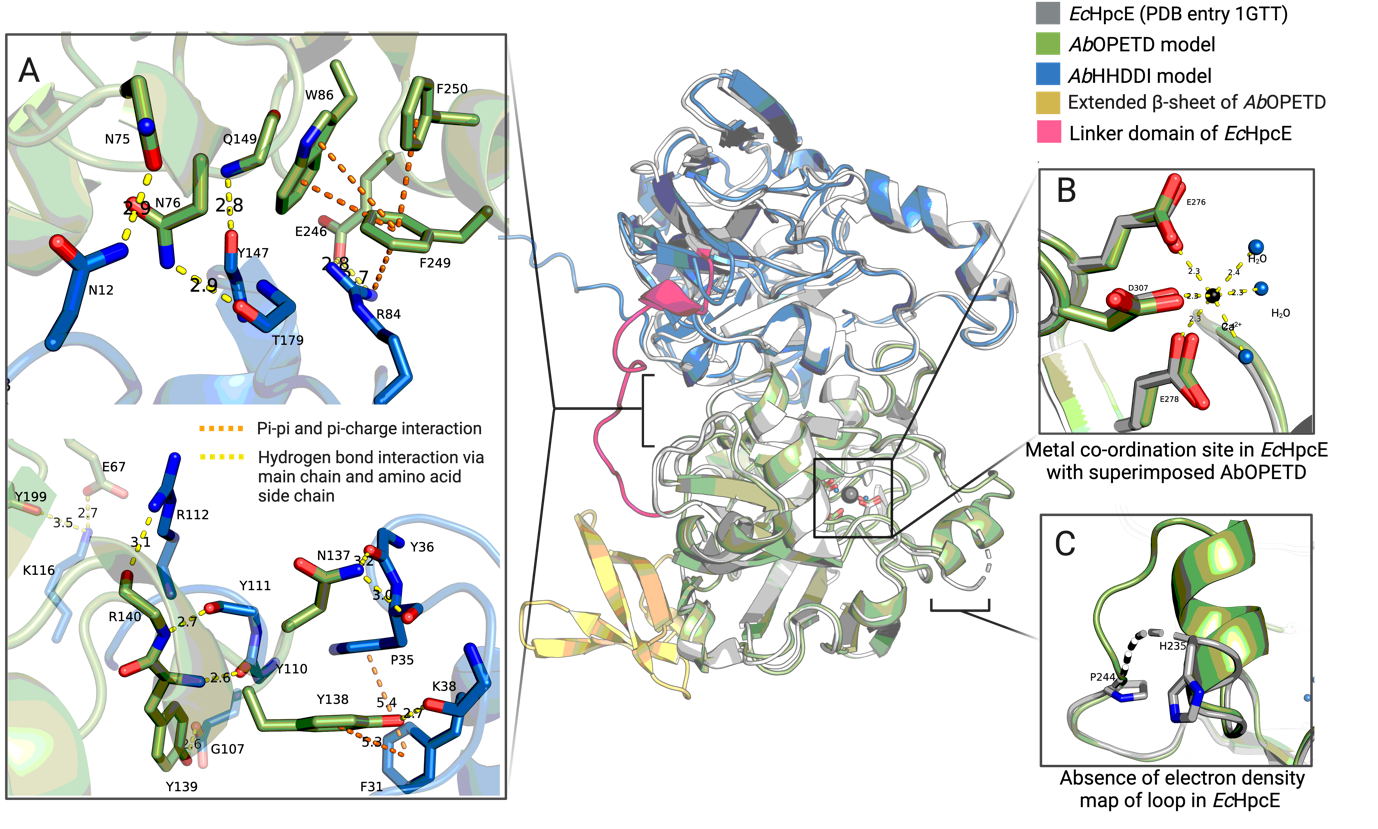
Figure S8. Superimposition structures of *Ab*OPETD and *Ab*HHDDI with *Ec*HpcE.** (A) The possible interactions between the monomeric *Ab*OPETD and *Ab*HHDDI on their surfaces are indicated by the orange and yellow dashes, representing hydrophobic interactions and hydrogen bonding, respectively. (B) The metal coordination feature of *Ec*HpcE, which involves a triad of negatively charged glutamate and aspartate, is observed in *Ab*OPETD. (C) The blind electron density map of the protein structure observes a loop region in *Ec*HpcE which *Ab*OPETD forms a loop with a short helix instead.


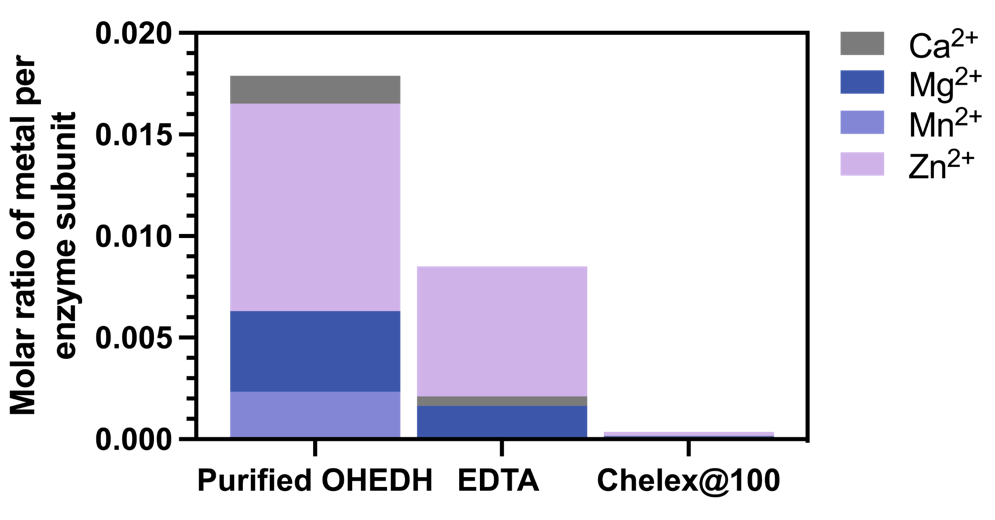


**Figure S9. Metal analysis by optical emission spectroscopy of OHEDH.** The molar ratios of M^2+^ ion per enzyme subunit of OHEDH after being treated with different types of chelating methods for preparing apo-OHEDH.

**
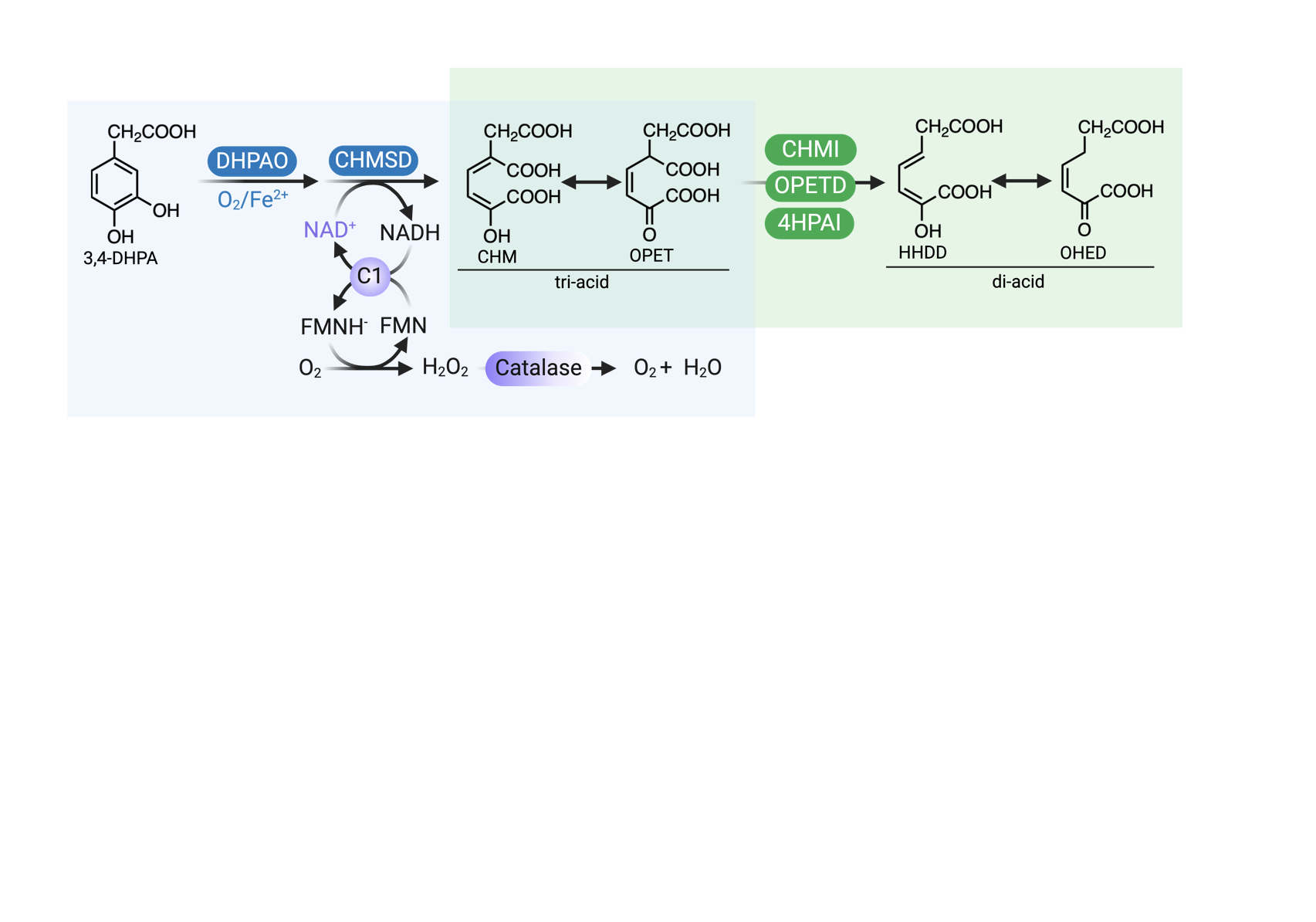
****Figure S10. The schematic representation of the one-pot enzymatic reaction to synthesis of tri-acid (CHM/OPET) and di-acid (HHDD/OHED) product intermediates with addition of two auxiliary systems**. The auxiliary systems implemented in the reaction include the C1-reductase reaction serving a regenerating NAD^+^ cofactor, and the catalase reaction scavenging hydrogen peroxide generated from the C1-reductase reaction.


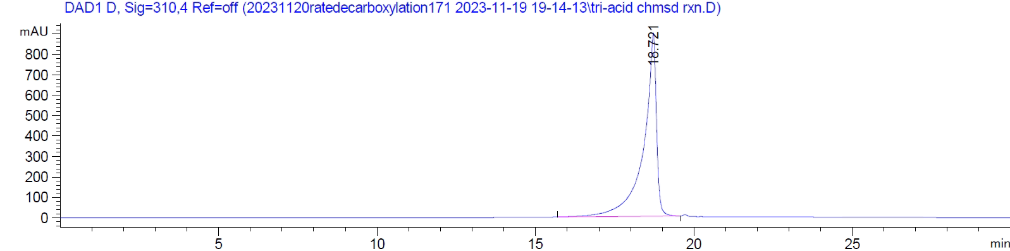


A


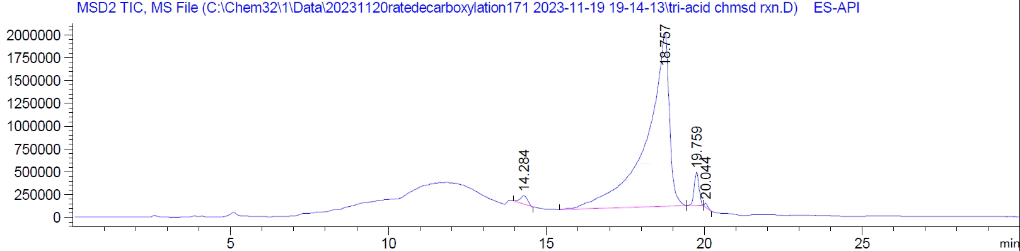


B


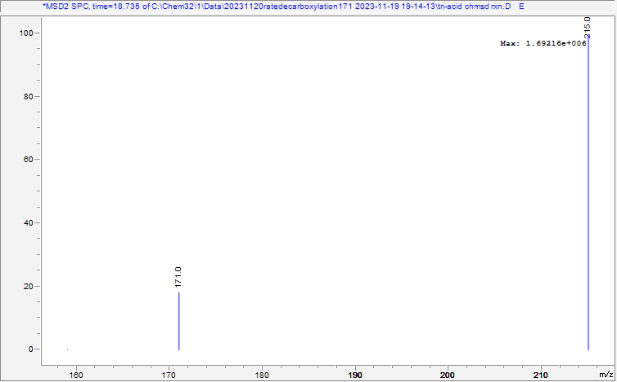

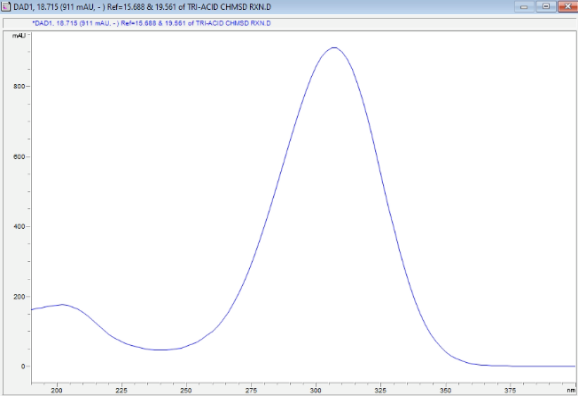


D

C

**Figure S11.** **The spectroscopic characteristics of partially purified tri-acid compounds.** (A) The HPLC chromatogram of tri-acid compounds detected at 310 nm. (B) Total ion chromatograms monitored by mass spectrometer. (C) The relative abundance and specific m/z 215.0 of tri-acid compounds. (D) The UV-visible absorption spectrum of tri-acid compounds.


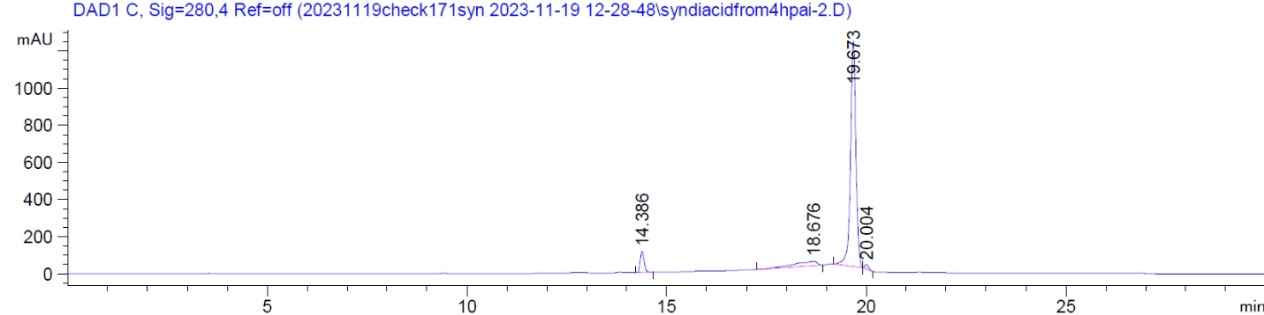


A

B

C


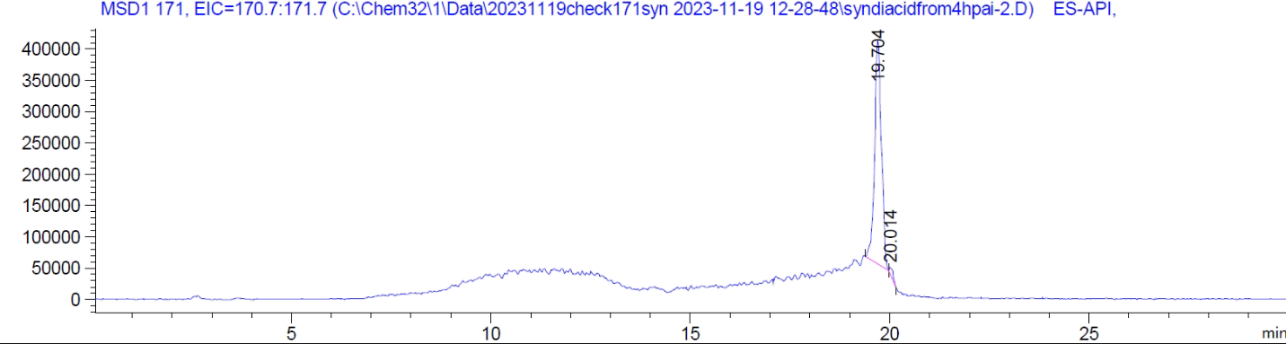


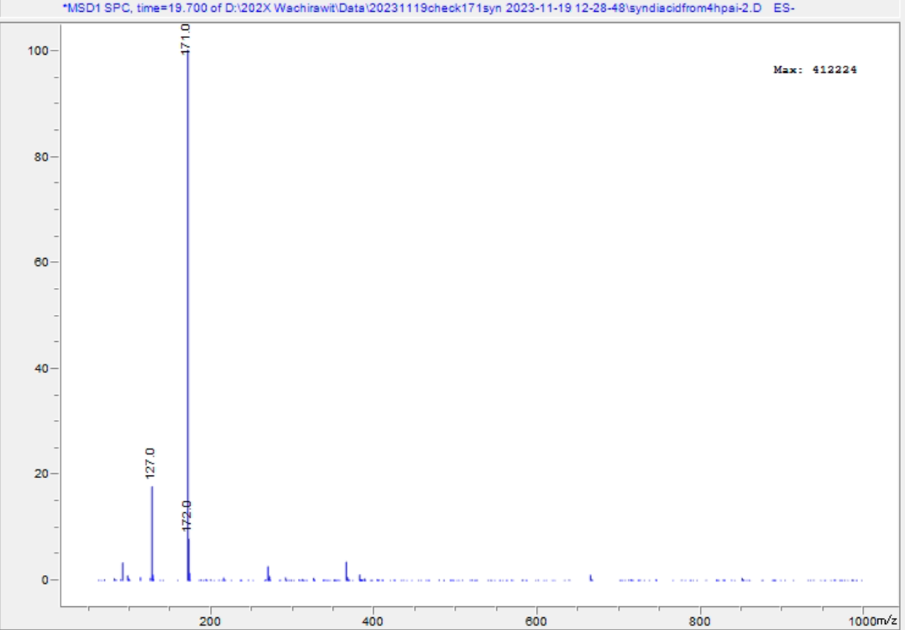


**Figure S12. The spectroscopic characteristics of partially purified di-acid compounds.** (A) The HPLC chromatogram of di-acid compounds monitored at 280 nm. (B) The selective ion chromatograms monitored by mass spectrometer. (C) The relative abundance and specific m/z 171 of di-acid compounds.

**
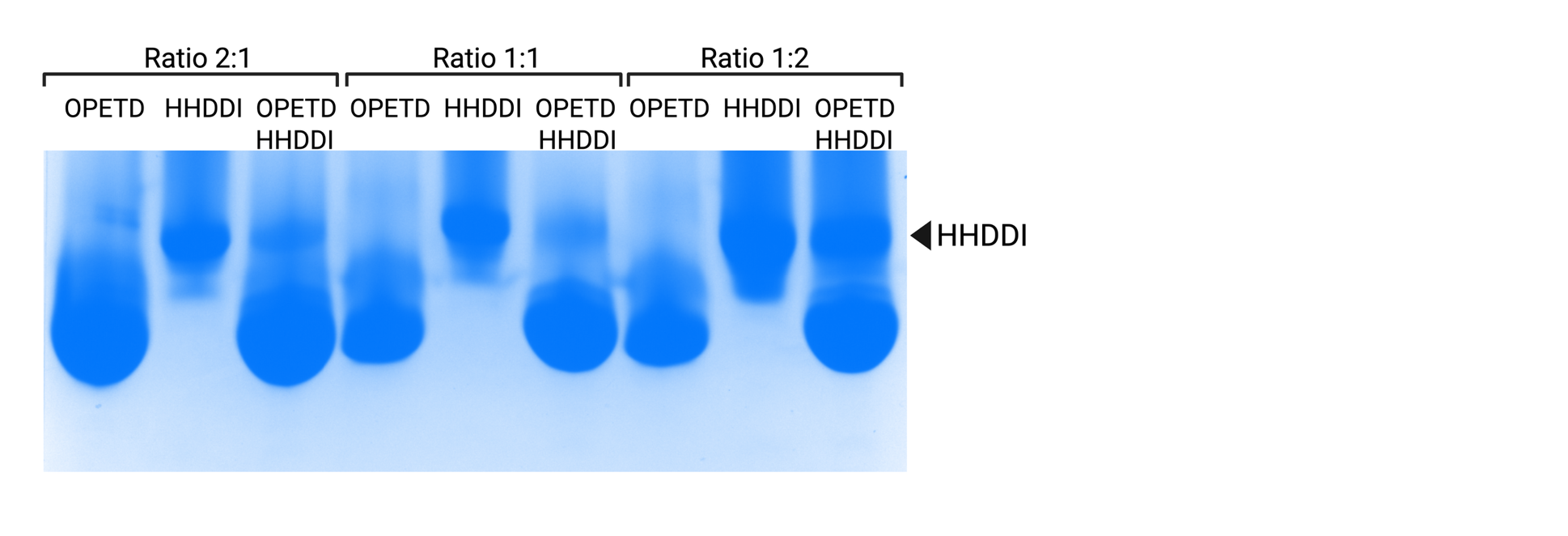
**

**Figure S13. Native-PAGE analysis for examining the protein-protein interactions of enzymes involved in the tri-acid decarboxylation of OPETD and HHDDI.** The OPETD and HHDDI proteins were pre-incubated at 4ºC in different ratios: 2:1, 1:1, and 1:2 before being loaded onto the Native-PAGE.
